# How well does NamSor perform in predicting the country of origin and ethnicity of individuals based on their first and last names?

**DOI:** 10.1371/journal.pone.0294562

**Published:** 2023-11-16

**Authors:** Paul Sebo

**Affiliations:** University Institute for Primary Care (IuMFE), University of Geneva, Geneva, Switzerland; The University of Hong Kong, HONG KONG

## Abstract

**Background:**

We aimed to evaluate NamSor’s performance in predicting the country of origin and ethnicity of individuals based on their first/last names.

**Methods:**

We retrieved the name and country of affiliation of all authors of PubMed publications in 2021, affiliated with universities in the twenty-two countries whose researchers authored ≥1,000 medical publications and whose percentage of migrants was <2.5% (N = 88,699). We estimated with NamSor their most likely "continent of origin" (Asia/Africa/Europe), "country of origin" and "ethnicity". We also examined two other variables that we created: “continent#2” ("Europe" replaced by "Europe/America/Oceania") and “country#2” ("Spain" replaced by “Spain/Hispanic American country” and "Portugal" replaced by "Portugal/Brazil"). Using "country of affiliation" as a proxy for "country of origin", we calculated for these five variables the proportion of misclassifications (= errorCodedWithoutNA) and the proportion of non-classifications (= naCoded). We repeated the analyses with a subsample consisting of all results with inference accuracy ≥50%.

**Results:**

For the full sample and the subsample, errorCodedWithoutNA was 16.0% and 12.6% for “continent”, 6.3% and 3.3% for “continent#2”, 27.3% and 19.5% for “country”, 19.7% and 11.4% for “country#2”, and 20.2% and 14.8% for “ethnicity”; naCoded was zero and 18.0% for all variables, except for “ethnicity” (zero and 10.7%).

**Conclusion:**

NamSor is accurate in determining the continent of origin, especially when using the modified variable (continent#2) and/or restricting the analysis to names with accuracy ≥50%. The risk of misclassification is higher with country of origin or ethnicity, but decreases, as with continent of origin, when using the modified variable (country#2) and/or the subsample.

## Introduction

Individuals are regularly discriminated against, for example because of their gender, their sexual orientation, their religion or their social or ethnic origin. The world of research is only a mirror of our society and does not escape these rejection behaviors. The study of discrimination in research mainly focused on gender inequalities, and numerous publications highlighted the major obstacles faced by women throughout their careers [1–6]. As a result, programs were launched in many countries to increase the representation of women in key academic positions and improve their career prospects [7].

However, rejection behaviors can be related to other social categories in addition to gender. The origin of researchers seems to be a criterion of discrimination according to several recent publications. Researchers from low- and middle-income countries (LMICs), for example, were found to be underrepresented as authors of articles [8–10] or as members of editorial boards [11].

To save time and resources, researchers can rely on NamSor, an online onomastic tool that infers origin from first and last names. NamSor combines three main advantages that are valuable to researchers: it is fast, cost-effective, and can be applied retroactively to large datasets. The methodology used by the algorithm to determine the most likely origin of individuals is relatively opaque to non-specialists, but likely relies on large databases combining names with cultural, ethnic and linguistic backgrounds.

NamSor can be particularly useful in bibliometric studies to determine country of origin or ethnicity when this variable is not available, whether to explore cross-cultural differences in research, inequalities in publications or citations of scientific articles related to the origin of researchers, reviewers or editors, or more broadly for any study including origin as a variable of interest. Indeed, such studies usually require large datasets and self-determination of country of origin or ethnicity is often not possible.

The tool was already used in several studies to estimate the origin of individuals. In a study comparing the number of citations, a proxy for scientific impact and relevance, for 13,000 articles published between 2015 and 2019 in fourteen high-impact general medical journals, we found that articles by first/last authors with African names were cited less often than other articles [10]. In another study evaluating ethnic and gender disparities in 442 prize presentation sessions at two prestigious surgical conferences in the UK over a 21-year period, the authors showed that almost half of the presenters (48%) were white men, followed by Asian men (25%) [12]. By contrast, there was only one black woman, one black man, and sixteen Asian women during these twenty-one years.

NamSor can help to determine both the gender and the origin of individuals. Its performance is high for gender inference, as demonstrated recently in a study comparing several gender detection tools [13], but, to our knowledge, there is no published data on the accuracy of this tool for determining the origin of individuals.

Based on a database of scientific publications (PubMed) including authors’ names and affiliations, the objective of this study was to evaluate the performance of NamSor for estimating the origin of individuals. Thanks to the progress made in data mining techniques, it is hypothesized that its performance should be relatively high but should vary according to individuals’ countries of origin.

## Methods

### Selection of publications and their authors

We used data from SCImago Journal & Country Rank to retrieve all countries whose researchers authored at least 1,000 scientific publications in 2020 in the field of medicine. SCImago Journal & Country Rank is a publicly available portal that includes scientific indicators for journals and countries developed from information in the Scopus® database [14]. Citation data are from over 34,000 titles and over 5,000 international publishers. Seventy-five countries met the inclusion criterion for the study, as shown in Table 1 (country #1: USA with 277,130 publications, country #75: Cuba with 1,059 publications). We also used data from International Migrant Stock 2020, available on the United Nations Population Division portal, to obtain the percentage of migrants by country in 2020. Data on estimates of the number (or "stock") of international migrants are presented as a percentage of the total population, by age, sex, and country of destination, and are based on national statistics, in most cases obtained from population censuses [15]. We selected the 22 countries for which this proportion was below 2.5 percent (Table 1). We restricted the study to these countries only in order to obtain names of researchers that were as homogeneous as possible and representative of the selected countries. The proportion of migrants for these countries ranged from zero for Cuba to 2.2 percent for Japan and Poland.

**Table 1:** List of countries whose researchers authored at least 1,000 medical publications in 2020, and percentage of migrants per country in 2020.

Rank	Country	Region	Number of medical publications in 2020	Migrant stock in 2020 (as a percentage of the total population)	Country included in the study^1^ (Y/N)
1	United States	Northern America	277130	15.3	N
2	China	Asiatic Region	172201	0.1	Y
3	United Kingdom	Western Europe	81178	13.8	N
4	Germany	Western Europe	62063	18.8	N
5	Italy	Western Europe	56413	10.6	N
6	Japan	Asiatic Region	48994	2.2	Y
7	Canada	Northern America	46214	21.3	N
8	India	Asiatic Region	44586	0.4	Y
9	Australia	Pacific Region	41640	30.1	N
10	France	Western Europe	41039	13.1	N
11	Spain	Western Europe	37726	14.6	N
12	Brazil	Latin America	30269	0.5	Y
13	Netherlands	Western Europe	29362	13.8	N
14	South Korea	Asiatic Region	28892	3.4	N
15	Turkey	Middle East	21840	7.2	N
16	Switzerland	Western Europe	21612	28.8	N
17	Iran	Middle East	21577	3.3	N
18	Russian Federation	Eastern Europe	17909	8.0	N
19	Sweden	Western Europe	17054	19.8	N
20	Belgium	Western Europe	14610	17.3	N
21	Poland	Eastern Europe	13993	2.2	Y
22	Denmark	Western Europe	12879	12.4	N
23	Taiwan	Asiatic Region	12421	N/A	N
24	Austria	Western Europe	10245	19.3	N
25	Egypt	Africa/Middle East	9639	0.5	Y
26	Mexico	Latin America	9347	0.9	Y
27	Saudi Arabia	Middle East	9255	38.6	N
28	Portugal	Western Europe	9145	9.8	N
29	Israel	Middle East	8733	22.6	N
30	South Africa	Africa	8432	4.8	N
31	Greece	Western Europe	8384	12.9	N
32	Norway	Western Europe	8292	15.7	N
33	Pakistan	Asiatic Region	7620	1.5	Y
34	Singapore	Asiatic Region	7458	43.1	N
35	Ireland	Western Europe	7040	17.6	N
36	Thailand	Asiatic Region	6610	5.2	N
37	Malaysia	Asiatic Region	6511	10.7	N
38	Finland	Western Europe	6452	7.0	N
39	Hong Kong	Asiatic Region	6325	39.5	N
40	New Zealand	Pacific Region	6201	28.7	N
41	Czech Republic	Eastern Europe	6082	5.1	N
42	Indonesia	Asiatic Region	5565	0.1	Y
43	Argentina	Latin America	4901	5.0	N
44	Chile	Latin America	4734	8.6	N
45	Colombia	Latin America	4722	3.7	N
46	Nigeria	Africa	4138	0.6	Y
47	Hungary	Eastern Europe	3529	6.1	N
48	Romania	Eastern Europe	3378	3.7	N
49	Iraq	Middle East	3345	0.9	Y
50	Ethiopia	Africa	2899	0.9	Y
51	Bangladesh	Asiatic Region	2690	1.3	Y
52	Croatia	Eastern Europe	2455	12.9	N
53	Viet Nam	Asiatic Region	2378	0.1	Y
54	Serbia	Eastern Europe	2374	9.4	N
55	Ukraine	Eastern Europe	2330	11.4	N
56	United Arab Emirates	Middle East	2188	88.1	N
57	Lebanon	Middle East	2179	25.1	N
58	Tunisia	Africa	2023	0.5	Y
59	Slovenia	Eastern Europe	1905	13.4	N
60	Kenya	Africa	1880	2.0	Y
61	Slovakia	Eastern Europe	1872	3.6	N
62	Peru	Latin America	1819	3.7	N
63	Qatar	Middle East	1802	77.3	N
64	Morocco	Africa	1762	0.3	Y
65	Jordan	Middle East	1738	33.9	N
66	Nepal	Asiatic Region	1624	1.7	Y
67	Ghana	Africa	1512	1.5	Y
68	Bulgaria	Eastern Europe	1457	2.7	N
69	Philippines	Asiatic Region	1386	0.2	Y
70	Uganda	Africa	1346	3.8	N
71	Ecuador	Latin America	1162	4.4	N
72	Cyprus	Western Europe	1153	15.8	N
73	Tanzania	Africa	1123	0.7	Y
74	Lithuania	Eastern Europe	1119	5.3	N
75	Cuba	Latin America	1059	0	Y

^1^ We selected for the study the twenty-two countries whose researchers authored at least 1,000 medical publications in 2020 and whose percentage of migrants was <2.5% in 2020.

Then, using PyMed [16], a Python library that gives access to PubMed, we extracted all publications in 2021 with at least one author affiliated with a university or research institute located in the selected countries (N = 118,897). S1 Table shows the Python program used for authors with affiliations in China. The same procedure was followed for the other countries of affiliation.

We obtained a csv file in which the variable ’authors’ had the following form (example for a publication authored by three researchers):

[{‘lastname’ : ‘x’, ‘firstname’ : ‘x’, ‘initials’ : ‘x’, ‘affiliation’ : ‘x’}, {‘lastname’ : ‘y’, ‘firstname’ : ‘y’, ‘initials’ : ‘y’, ‘affiliation’ : ‘y’}, {‘lastname’ : ‘z’, ‘firstname’ : ‘z’, ‘initials’ : ‘z’, ‘affiliation’ : ‘z’}]

Using Stata, we created the variable ’author1’ (i.e., data for first authors only) and the variable ’country1’ (i.e., country of affiliation for first authors only). As the Python program retrieved all articles with at least one author affiliated with one of the countries selected for the study, we removed the publications for which the affiliation to the selected countries did not concern the first author. To do this, we used regular expressions (‘regexm’ in Stata) to extract the country of affiliation of the first author of each article. For missing data (i.e., publications for which the author’s country of affiliation was missing), we added a manual search using the information provided by PubMed (city or university name). Then, all publications for which the country of affiliation of the first author was not one of those selected for the study were removed from the database. The study database contained data for 88,699 publications. Since the authors of these publications were all affiliated with countries with relatively homogeneous populations, we used their country of affiliation as a proxy for their country of origin.

The database included authors with a single affiliation (N = 68,133) and authors with multiple affiliations (N = 20,566). This second group of researchers could possibly include authors with affiliations in several countries (e.g. USA and China). For these researchers, the country of affiliation used was the one that was part of the countries selected for the study (China and not the USA in the case above). We compared the results of the study using the full sample and the subsample consisting only of authors with a single affiliation to assess if the procedure followed for authors with multiple affiliations was appropriate. We again used regular expressions to identify the two groups of researchers. For a researcher with at least two affiliations, these affiliations were separated in the csv file created with the Python program by the newline character ‘\n’ or a semicolon.

### NamSor Applied Onomastics

The authors’ names were classified with NamSor Applied Onomastics, a name recognition software [17]. The software recognizes the linguistic or cultural origin of each name and assigns a gender (male or female) and/or an onomastic class (e.g., China or India). As the estimation is probabilistic, the software also provides a probability for the inference (‘probabilityCalibrated’) ranging from zero to one, with ’one’ meaning 100% accuracy. The way this parameter is calculated is described elsewhere [18].

NamSor operates on the principles of linguistic analysis, probabilistic modeling, and machine learning. By leveraging a vast and diverse database of names from around the world, NamSor identifies patterns in names associated with specific regions and ethnicities. It harnesses linguistic attributes, including phonetics and morphology, to enhance its recognition capabilities. In addition, machine learning techniques empower NamSor to continually improve its accuracy through learning from its database. However, the tool has certain potential limitations. First, names can be highly diverse and may not always accurately reflect an individual’s true origin. For example, people may have names from different cultures due to migration, intercultural marriages, or other factors. Second, the algorithm’s performance can vary significantly by country and ethnicity. It may work very well for some regions but less effectively for others. Third, in cases where a person has a first name from one region and a last name from another, the tool may not perform optimally. Finally, NamSor may not be well-suited for areas with highly diverse populations, such as multicultural urban centers or regions with extensive international immigration. In such places, the algorithm’s accuracy may be challenged.

Names can be classified by NamSor in three different ways: by continent of origin (only three continents: Asia, Africa or Europe), country of origin (e.g., China or India) or ethnicity (e.g., Chinese or Indian). The origin of a name refers to the country or continent where an individual was born, or where the individual’s parents or ancestors came from. According to NamSor, the most likely country of origin for the name “Keith Haring” is the United Kingdom with a probability of 48% (i.e., probabilityCalibrated = 0.48). An ethnicity (or an ethnic group) is a group of individuals who identify with each other on the basis of shared attributes distinguishing them from other groups, such as common sets of cultural traditions, ancestry, language and religion. According to NamSor, the most likely ethnicity for the name “Keith Haring” is German with a probability of 61% (i.e., probabilityCalibrated = 0.61). NamSor can also classify names according to race, a classification that we did not use in our study. This categorization includes six classes and is based on the taxonomy used for the US census: White, Black or African American, American Indian or Alaska Native, Asian, Hispanic or Latino, and Native Hawaiian or other Pacific Islander. According to NamSor, "Keith Haring" is most likely "White" (probability = 73%).

We created two other variables: continent#2 ("Europe" replaced by "Europe, America or Oceania") and country#2 ("Spain" replaced by “Spain or Hispanic American country” and "Portugal" replaced by "Portugal or Brazil"). We added these variables because a preliminary analysis of our data showed that a majority of researchers with Hispanic or Portuguese names who were affiliated with universities or research institutes in Brazil, Mexico or Cuba were considered to be from either Spain or Portugal.

### Performance analysis

We evaluated NamSor’s performance by computing three efficiency metrics. These metrics refer to the confusion matrix that contains three components, with ’c’ corresponding to correct classifications, ’i’ to misclassifications (i.e., a wrong continent, country or ethnicity assigned to a name) and ’u’ to non-classifications (i.e., no continent, country or ethnicity assigned). In this study, we considered the “actual country of origin” of the researchers to be their country of affiliation, as extracted from the database listing the authors of publications. The “predicted country of origin” was the country determined by NamSor using the researchers’ first and last names. These definitions also apply to continent of origin and ethnicity.

**Table:** 

	Correct continent, country or ethnicity (predicted)	Incorrect continent, country or ethnicity (predicted)	Unknown (predicted)
Continent, country or ethnicity (actual)	c	i	u

*errorCoded* = (i + u) / (c + i + u)

*errorCodedWithoutNA* = (i) / (c + i)

*naCoded* = (u) / (c + i + u)

The three performance metrics computed in the study can be interpreted as follows: *errorCoded* estimates the proportion of misclassifications and non-classifications (this measure therefore penalizes both types of errors equally), *errorCodedWithoutNA* measures the proportion of misclassifications excluding non-classifications, and *naCoded* measures the proportion of non-classifications. The same metrics were computed in several recent studies, including some conducted by our research team, to estimate the performance of gender detection tools [13, 18–20]. These tools allow to determine the gender of individuals based on their name.

We repeated the analyses by removing all results with inference accuracy <40% (i.e., probabilityCalibrated <0.4), <50% (i.e., probabilityCalibrated <0.5), <60% (i.e., probabilityCalibrated <0.6) and <70% (i.e., probabilityCalibrated <0.7), respectively. All assignments made with an accuracy level below the selected threshold value were considered as non-classifications. These sensitivity analyses were conducted to determine how the proportion of misclassifications and the proportion of non-classifications changed as the accuracy threshold increased or decreased.

### Estimating the proportion of foreign researchers in the study sample

We used the misclassification proportion for "country#2", restricting the analysis to only those researchers for whom the country of origin was determined with an inference accuracy ≥70%, as an indicator of the proportion of foreign researchers in the study sample. Indeed, the level of misclassification can be considered as an indirect measure of the maximum proportion of foreign researchers according to the following formula: "proportion of misclassification with inference accuracy ≥70%" = "proportion of misclassification due to NamSor error" + "proportion of misclassification due to foreign researchers". Even including in the calculation only those researchers for whom their country of origin was determined with an accuracy ≥70%, the proportion of foreign researchers should in fact be lower than the proportion of misclassification for “country#2”, since NamSor is not 100% accurate. As researchers are expected to be more mobile than the general population, we hypothesize that by limiting the countries of affiliation to only those countries with a migrant stock <2.5% the proportion of foreign researchers would be at most 5% in the study. We performed all analyses with STATA version 15.1 (College Station, TX, USA).

### Ethical considerations

Since this study did not involve the collection of personal health-related data it did not require ethical review, according to current Swiss law.

## Results

The main results of the study are presented in Tables 2–4, for the full sample, for authors with a single affiliation, and for the four subsamples including only names for which the inference accuracy was, respectively, ≥40%, ≥50%, ≥60%, and ≥70%. Table 2 shows for each of the twenty-two selected countries the number of researchers whose name origin was correctly classified by NamSor. These data are then summarized in Table 3 (confusion matrices) and Table 4 (performance metrics).

**Table 2:** Number of researchers whose name origin, sorted by continent, country and ethnicity, was correctly classified by NamSor (N = 88,699 researchers from twenty-two countries).

Country of affiliation of researchers, ranked by number of medical publications in 2020	Continent, number of data	Continent, number (%) of correctly classified names	Country, number of data	Country, number (%) of correctly classified names	Ethnicity, number of data	Ethnicity, number (%) of correctly classified names
China		Asia		China		Chinese
Full sample	7702	7462 (96.9)	7702	5837 (75.8)	7702	6506 (84.5)
Authors with a single affiliation	5330	5182 (97.2)	5330	4035 (75.7)	5330	4483 (84.1)
Accuracy ≥40%	7516	7290 (97.0)	7516	5772 (76.8)	7550	6461 (85.6)
Accuracy ≥50%	6047	5882 (97.3)	6047	4862 (80.4)	7421	6383 (86.0)
Accuracy ≥60%	5369	5226 (97.3)	5369	4312 (80.3)	7237	6253 (86.4)
Accuracy ≥70%	4554	4434 (97.4)	4554	3646 (80.1)	6874	5953 (86.6)
Japan		Asia		Japan		Japanese
Full sample	6362	6096 (95.8)	6362	5451 (85.7)	6362	5430 (85.4)
Authors with a single affiliation	4930	4751 (96.4)	4930	4275 (86.7)	4930	4266 (86.5)
Accuracy ≥40%	6308	6067 (96.2)	6308	5443 (86.3)	6252	5417 (86.6)
Accuracy ≥50%	6032	5878 (97.5)	6032	5374 (89.1)	6197	5412 (87.3)
Accuracy ≥60%	5905	5785 (98.0)	5905	5320 (90.1)	6125	5390 (88.0)
Accuracy ≥70%	5732	5636 (98.3)	5732	5223 (91.1)	6026	5350 (88.8)
India		Asia		India		Indian
Full sample	5362	4698 (87.6)	5362	3406 (63.5)	5362	4307 (80.3)
Authors with a single affiliation	4749	4171 (87.8)	4749	3022 (63.6)	4749	3820 (80.4)
Accuracy ≥40%	5106	4537 (88.9)	5106	3325 (65.1)	5070	4213 (83.1)
Accuracy ≥50%	3371	3177 (94.3)	3371	2530 (75.1)	4885	4139 (84.7)
Accuracy ≥60%	2652	2542 (95.9)	2652	2066 (77.9)	4638	3993 (86.1)
Accuracy ≥70%	1916	1857 (96.9)	1916	1521 (79.4)	4291	3748 (87.4)
Brazil^1^		Europe		Portugal		Portuguese
Full sample	2829	2666 (94.2)	2829	1635 (57.8)	2829	1790 (63.3)
Authors with a single affiliation	2140	2019 (94.4)	2140	1283 (60.0)	2140	1381 (64.5)
Accuracy ≥40%	2724	2584 (94.9)	2724	1610 (59.1)	2617	1737 (66.4)
Accuracy ≥50%	2098	2032 (96.9)	2098	1429 (68.1)	2480	1685 (67.9)
Accuracy ≥60%	1811	1766 (97.5)	1811	1317 (72.7)	2281	1615 (70.8)
Accuracy ≥70%	1520	1491 (98.1)	1520	1162 (76.5)	2093	1537 (73.4)
Poland		Europe		Poland		Polish
Full sample	18441	18106 (98.2)	18441	16816 (91.2)	18441	16466 (89.3)
Authors with a single affiliation	16152	15906 (98.5)	16152	14887 (92.2)	16152	14564 (90.2)
Accuracy ≥40%	18168	17862 (98.3)	18168	16731 (92.1)	17744	16245 (91.6)
Accuracy ≥50%	16613	16401 (98.7)	16613	15814 (95.2)	17287	16026 (92.7)
Accuracy ≥60%	15744	15564 (98.9)	15744	15136 (96.1)	16676	15675 (94.0)
Accuracy ≥70%	14761	14619 (99.0)	14761	14313 (97.0)	15885	15057 (94.8)
Egypt		Africa		Egypt		Egyptian
Full sample	9476	8840 (93.3)	9476	8615 (90.9)	9476	7677 (81.0)
Authors with a single affiliation	6798	6372 (93.7)	6798	6214 (91.4)	6798	5578 (82.1)
Accuracy ≥40%	9280	8726 (94.0)	9280	8541 (92.0)	8783	7448 (84.8)
Accuracy ≥50%	8145	7928 (97.3)	8145	7889 (96.9)	8372	7282 (87.0)
Accuracy ≥60%	7466	7346 (98.4)	7466	7325 (98.1)	7842	6986 (89.1)
Accuracy ≥70%	6631	6560 (98.9)	6631	6553 (98.8)	7184	6587 (91.7)
Mexico^2^		Europe		Spain		Hispanic
Full sample	5868	5595 (95.4)	5868	4845 (82.6)	5868	4800 (81.8)
Authors with a single affiliation	4680	4471 (95.5)	4680	3883 (83.0)	4680	3858 (82.4)
Accuracy ≥40%	5719	5480 (95.8)	5719	4802 (84.0)	5179	4435 (85.6)
Accuracy ≥50%	4629	4527 (97.8)	4629	4199 (90.7)	4686	4101 (87.5)
Accuracy ≥60%	4068	3995 (98.2)	4068	3792 (93.2)	3991	3576 (89.6)
Accuracy ≥70%	3475	3424 (98.5)	3475	3295 (94.8)	3031	2751 (90.8)
Pakistan		Asia		Pakistan		Pakistanis
Full sample	6810	6674 (98.0)	6810	6388 (93.8)	6810	5882 (86.4)
Authors with a single affiliation	5644	5534 (98.1)	5644	5294 (93.8)	5644	4862 (86.1)
Accuracy ≥40%	6744	6626 (98.3)	6744	6367 (94.4)	6507	5787 (88.9)
Accuracy ≥50%	6202	6160 (99.3)	6202	6035 (97.3)	6327	5690 (89.9)
Accuracy ≥60%	5872	5851 (99.6)	5872	5777 (98.4)	6132	5587 (91.1)
Accuracy ≥70%	5404	5387 (99.7)	5404	5340 (98.8)	5852	5381 (92.0)
Indonesia		Asia		Indonesia		Indonesian
Full sample	3828	3403 (88.9)	3828	2980 (77.9)	3828	2820 (73.7)
Authors with a single affiliation	2609	2317 (88.8)	2609	2044 (78.3)	2609	1937 (74.2)
Accuracy ≥40%	3692	3339 (90.4)	3692	2935 (79.5)	3397	2717 (80.0)
Accuracy ≥50%	3017	2883 (95.6)	3017	2644 (87.6)	3178	2634 (82.9)
Accuracy ≥60%	2732	2654 (97.1)	2732	2489 (91.1)	2948	2536 (86.0)
Accuracy ≥70%	2451	2411 (98.4)	2451	2291 (93.5)	2679	2366 (88.3)
Nigeria		Africa		Nigeria		Nigerian
Full sample	3370	3104 (92.1)	3370	2553 (75.8)	3370	2547 (75.6)
Authors with a single affiliation	2238	2086 (93.2)	2238	1746 (78.0)	2238	1743 (77.9)
Accuracy ≥40%	3265	3018 (92.4)	3265	2522 (77.2)	3044	2481 (81.5)
Accuracy ≥50%	2695	2579 (95.7)	2695	2352 (87.3)	2899	2427 (83.7)
Accuracy ≥60%	2493	2400 (96.3)	2493	2258 (90.6)	2721	2352 (86.4)
Accuracy ≥70%	2272	2214 (97.5)	2272	2129 (93.7)	2505	2241 (89.5)
Iraq		Asia		Iraq		Iraqi
Full sample	1006	829 (82.4)	1006	270 (26.8)	1006	247 (24.6)
Authors with a single affiliation	737	603 (81.8)	737	208 (28.2)	737	191 (25.9)
Accuracy ≥40%	903	742 (82.2)	903	249 (27.6)	771	212 (27.5)
Accuracy ≥50%	507	436 (86.0)	507	171 (33.7)	661	194 (29.4)
Accuracy ≥60%	335	286 (85.4)	335	129 (38.5)	513	154 (30.0)
Accuracy ≥70%	225	195 (86.7)	225	95 (42.2)	391	119 (30.4)
Ethiopia		Africa		Ethiopia		Ethiopian
Full sample	4030	3861 (95.8)	4030	3671 (91.1)	4030	3451 (85.6)
Authors with a single affiliation	3348	3210 (95.9)	3348	3057 (91.3)	3348	2872 (85.8)
Accuracy ≥40%	3960	3808 (96.2)	3960	3653 (92.3)	3795	3387 (89.3)
Accuracy ≥50%	3685	3606 (97.9)	3685	3556 (96.5)	3671	3335 (90.9)
Accuracy ≥60%	3589	3546 (98.8)	3589	3516 (98.0)	3513	3242 (92.3)
Accuracy ≥70%	3489	3466 (99.3)	3489	3448 (98.8)	3359	3130 (93.2)
Bangladesh		Asia		Bangladesh		Bangladeshi
Full sample	2491	2420 (97.2)	2491	1955 (78.5)	2491	1805 (72.5)
Authors with a single affiliation	1724	1685 (97.7)	1724	1338 (77.6)	1724	1230 (71.4)
Accuracy ≥40%	2445	2383 (97.5)	2445	1941 (79.4)	2328	1765 (75.8)
Accuracy ≥50%	2054	2033 (99.0)	2054	1784 (86.9)	2232	1726 (77.3)
Accuracy ≥60%	1866	1855 (99.4)	1866	1697 (90.9)	2096	1656 (79.0)
Accuracy ≥70%	1667	1662 (99.7)	1667	1565 (93.9)	1934	1576 (81.5)
Vietnam		Asia		Vietnam		Vietnamese
Full sample	1959	1894 (96.7)	1959	1842 (94.0)	1959	1809 (92.3)
Authors with a single affiliation	1043	1017 (97.5)	1043	1005 (96.4)	1043	987 (94.6)
Accuracy ≥40%	1955	1894 (96.9)	1955	1842 (94.2)	1942	1804 (92.9)
Accuracy ≥50%	1923	1885 (98.0)	1923	1837 (95.5)	1922	1793 (93.3)
Accuracy ≥60%	1905	1876 (98.5)	1905	1833 (96.2)	1895	1779 (93.9)
Accuracy ≥70%	1889	1864 (98.7)	1889	1828 (96.8)	1854	1752 (94.5)
Tunisia		Africa		Tunisia		Tunisian
Full sample	1632	1589 (97.4)	1632	1224 (75.0)	1632	1072 (65.7)
Authors with a single affiliation	987	957 (97.0)	987	719 (72.9)	987	651 (66.0)
Accuracy ≥40%	1547	1512 (97.7)	1547	1195 (77.3)	1452	1018 (70.1)
Accuracy ≥50%	1103	1091 (98.9)	1103	999 (90.6)	1351	975 (72.2)
Accuracy ≥60%	912	908 (99.6)	912	853 (93.5)	1181	896 (75.9)
Accuracy ≥70%	720	716 (99.4)	720	684 (95.0)	995	798 (80.2)
Kenya		Africa		Kenya		Kenyan
Full sample	1187	972 (81.9)	1187	665 (56.0)	1187	591 (49.8)
Authors with a single affiliation	647	545 (84.2)	647	368 (56.9)	647	325 (50.2)
Accuracy ≥40%	1153	953 (82.7)	1153	657 (57.0)	959	561 (58.5)
Accuracy ≥50%	835	713 (85.4)	835	582 (69.7)	878	545 (62.1)
Accuracy ≥60%	732	646 (88.3)	732	545 (74.5)	793	516 (65.1)
Accuracy ≥70%	629	572 (90.9)	629	489 (77.7)	707	489 (69.2)
Morocco		Africa		Morocco		Moroccan
Full sample	1545	1469 (95.1)	1545	1091 (70.6)	1545	809 (52.4)
Authors with a single affiliation	1036	993 (95.9)	1036	723 (69.8)	1036	528 (51.0)
Accuracy ≥40%	1466	1396 (95.2)	1466	1048 (71.5)	1234	706 (57.2)
Accuracy ≥50%	934	914 (97.9)	934	789 (84.5)	1087	641 (59.0)
Accuracy ≥60%	752	743 (98.8)	752	660 (87.8)	893	544 (60.9)
Accuracy ≥70%	570	563 (98.8)	570	507 (89.0)	686	429 (62.5)
Nepal		Asia		Nepal		Nepalese
Full sample	1327	1196 (90.1)	1327	406 (30.6)	1327	900 (67.8)
Authors with a single affiliation	1139	1033 (90.7)	1139	361 (31.7)	1139	788 (69.2)
Accuracy ≥40%	1209	1102 (91.2)	1209	383 (31.7)	1239	854 (68.9)
Accuracy ≥50%	655	613 (93.6)	655	233 (35.6)	1168	813 (69.6)
Accuracy ≥60%	436	409 (93.8)	436	151 (34.6)	1054	734 (69.6)
Accuracy ≥70%	271	254 (93.7)	271	87 (32.1)	914	625 (68.4)
Ghana		Africa		Ghana		Ghanaian
Full sample	1383	1251 (90.5)	1383	1036 (74.9)	1383	947 (68.5)
Authors with a single affiliation	867	788 (90.9)	867	650 (75.0)	867	600 (69.2)
Accuracy ≥40%	1349	1225 (90.8)	1349	1025 (76.0)	1205	909 (75.4)
Accuracy ≥50%	1098	1043 (95.0)	1098	945 (86.1)	1115	888 (79.6)
Accuracy ≥60%	1009	977 (96.8)	1009	905 (89.7)	1011	857 (84.8)
Accuracy ≥70%	917	893 (97.4)	917	839 (91.5)	941	824 (87.6)
Philippines		Asia		Philippines		Hispanic
Full sample	1113	141 (12.7)	1113	0	1113	421 (37.8)
Authors with a single affiliation	747	82 (11.0)	747	0	747	289 (38.7)
Accuracy ≥40%	1018	129 (12.7)	1018	0	795	342 (43.0)
Accuracy ≥50%	510	78 (15.3)	510	0	655	304 (46.4)
Accuracy ≥60%	357	64 (17.9)	357	0	495	261 (52.7)
Accuracy ≥70%	226	45 (19.9)	226	0	366	196 (53.6)
Tanzania		Africa		Tanzania		Tanzanian
Full sample	673	544 (80.8)	673	293 (43.5)	673	291 (43.2)
Authors with a single affiliation	355	279 (78.6)	355	151 (42.5)	355	156 (43.9)
Accuracy ≥40%	617	503 (81.5)	617	272 (44.1)	529	257 (48.6)
Accuracy ≥50%	387	318 (82.2)	387	212 (54.8)	467	238 (51.0)
Accuracy ≥60%	307	248 (80.8)	307	177 (57.7)	402	222 (55.2)
Accuracy ≥70%	211	170 (80.6)	211	121 (57.4)	342	192 (56.1)
Cuba^2^		Europe		Spain		Hispanic
Full sample	305	296 (97.1)	305	261 (85.6)	305	243 (79.7)
Authors with a single affiliation	233	228 (97.9)	233	204 (87.6)	233	186 (79.8)
Accuracy ≥40%	296	288 (97.3)	296	256 (86.5)	279	226 (81.0)
Accuracy ≥50%	237	234 (98.7)	237	225 (94.9)	258	216 (83.7)
Accuracy ≥60%	220	218 (99.1)	220	212 (96.4)	219	191 (87.2)
Accuracy ≥70%	188	187 (99.5)	188	184 (97.9)	177	157 (88.7)

Data are presented for the full sample, for authors with a single affiliation, and for four subsamples including only names for which the inference accuracy was, respectively, ≥40%, ≥50%, ≥60% and ≥70%.

^1^ The table shows the number of names correctly classified for this country, after replacing for the variable "continent" the category "Europe" by the category "Europe, America or Oceania", and for the variable "country" the category "Portugal" by the category "Portugal or Brazil".

^2^ The table shows the number of names correctly classified for this country, after replacing for the variable "continent" the category "Europe" by the category "Europe, America or Oceania", and for the variable "country" the category "Spain" by the category "Spain or Hispanic American country".

**Table 3:** Confusion matrices for the origin of the names of 88,699 researchers.

Variable	Number (%) of correctly classified names	Number (%) of misclassified names	Number (%) of unclassified names
Full sample			
Continent of origin (Asia, Africa or Europe)	74549 (84.0)	14150 (16.0)	0
Continent#2^1^	83106 (93.7)	5593 (6.3)	0
Country of origin	64499 (72.7)	24200 (27.3)	0
Country#2^2^	71240 (80.3)	17459 (19.7)	0
Ethnicity	70811 (79.8)	17888 (20.2)	0
Authors with a single affiliation			
Continent of origin (Asia, Africa or Europe)	57511 (84.4)	10622 (15.6)	0
Continent#2^1^	64229 (94.3)	3904 (5.7)	0
Country of origin	50097 (73.5)	18036 (26.5)	0
Country#2^2^	55467 (81.4)	12666 (18.6)	0
Ethnicity	55295 (81.2)	12838 (18.8)	0
Accuracy of the inference ≥40%			
Continent of origin (Asia, Africa or Europe)	73112 (82.4)	13328 (15.0)	2259 (2.6)
Continent#2^1^	81464 (91.8)	4976 (5.6)	2259 (2.6)
Country of origin	63901 (72.0)	22539 (25.4)	2259 (2.6)
Country#2^2^	70569 (79.6)	15871 (17.8)	2559 (2.6)
Ethnicity	68982 (77.8)	13689 (15.4)	6028 (6.8)
Accuracy of the inference ≥50%			
Continent of origin (Asia, Africa or Europe)	63618 (71.7)	9159 (10.3)	15922 (18.0)
Continent#2^1^	70411 (79.4)	2366 (2.6)	15922 (18.0)
Country of origin	58608 (66.0)	14169 (16.0)	15922 (18.0)
Country#2^2^	64461 (72.7)	8316 (9.3)	15922 (18.0)
Ethnicity	67447 (76.0)	11750 (13.3)	9502 (10.7)
Accuracy of the inference ≥60%			
Continent of origin (Asia, Africa or Europe)	58926 (66.4)	7606 (8.6)	22167 (25.0)
Continent#2^1^	64905 (73.2)	1627 (1.8)	22167 (25.0)
Country of origin	55149 (62.2)	11383 (12.8)	22167 (25.0)
Country#2^2^	60470 (68.2)	6062 (6.8)	22167 (25.0)
Ethnicity	65015 (73.3)	9641 (10.9)	14043 (15.8)
Accuracy of the inference ≥70%			
Continent of origin (Asia, Africa or Europe)	53518 (60.3)	6200 (7.0)	28981 (32.7)
Continent#2^1^	58620 (66.1)	1098 (1.2)	28981 (32.7)
Country of origin	50679 (57.1)	9039 (10.2)	28981 (32.7)
Country#2^2^	55320 (62.3)	4398 (5.0)	28981 (32.7)
Ethnicity	61258 (69.1)	7828 (8.8)	19613 (22.1)

Data are presented for the full sample, for authors with a single affiliation, and for four subsamples including only names for which the inference accuracy was, respectively, ≥40%, ≥50%, ≥60% and ≥70%.

^1^ “Europe” replaced by “Europe, America or Oceania”

^2^ "Spain" replaced by “Spain or Hispanic American country” and "Portugal" replaced by "Portugal or Brazil”

**Table 4:** Performance metrics (i.e., errorCoded, errorCodedWithoutNA and naCoded) for the origin of the names of 88,699 researchers.

Variable	errorCoded^1^	errorCodedWithoutNA^2^	naCoded^3^
Full sample			
Continent of origin (Asia, Africa or Europe)	0.1595	0.1595	0
Continent#2^4^	0.0631	0.0631	0
Country of origin	0.2728	0.2728	0
Country#2^5^	0.1968	0.1968	0
Ethnicity	0.2017	0.2017	0
Authors with a single affiliation			
Continent of origin (Asia, Africa or Europe)	0.1559	0.1559	0
Continent#2^4^	0.0573	0.0573	0
Country of origin	0.2647	0.2647	0
Country#2^5^	0.1859	0.1859	0
Ethnicity	0.1884	0.1884	0
Accuracy of the inference ≥40%			
Continent of origin (Asia, Africa or Europe)	0.1757	0.1542	0.0255
Continent#2^4^	0.0816	0.0576	0.0255
Country of origin	0.2796	0.2608	0.0255
Country#2^5^	0.2044	0.1836	0.0255
Ethnicity	0.2223	0.1656	0.0680
Accuracy of the inference ≥50%			
Continent of origin (Asia, Africa or Europe)	0.2828	0.1259	0.1795
Continent#2^4^	0.2062	0.0325	0.1795
Country of origin	0.3393	0.1947	0.1795
Country#2^5^	0.2733	0.1143	0.1795
Ethnicity	0.2396	0.1484	0.1071
Accuracy of the inference ≥60%			
Continent of origin (Asia, Africa or Europe)	0.3357	0.1143	0.2499
Continent#2^4^	0.2683	0.0245	0.2499
Country of origin	0.3783	0.1711	0.2499
Country#2^5^	0.3183	0.0911	0.2499
Ethnicity	0.2670	0.1291	0.1583
Accuracy of the inference ≥70%			
Continent of origin (Asia, Africa or Europe)	0.3966	0.1038	0.3267
Continent#2^4^	0.3391	0.0184	0.3267
Country of origin	0.4286	0.1514	0.3267
Country#2^5^	0.3763	0.0737	0.3267
Ethnicity	0.3094	0.1133	0.2211

Data are presented for the full sample, for authors with a single affiliation, and for four subsamples including only names for which the inference accuracy was, respectively, ≥40%, ≥50%, ≥60% and ≥70%.

^1^ errorCoded = proportion of misclassifications (i.e., wrong continent, country or ethnicity assigned to a name) and non-classifications (i.e., no continent, country or ethnicity assigned)

^2^ errorCodedWithoutNA = proportion of misclassifications excluding non-classifications

^3^ naCoded = proportion of non-classifications

^4^ “Europe” replaced by “Europe, America or Oceania”

^5^ "Spain" replaced by “Spain or Hispanic American country” and "Portugal" replaced by "Portugal or Brazil”

In addition, S2 Table shows for each country of affiliation the countries of origin estimated by NamSor. These countries, five per country of affiliation, are ranked in the table by the number of inferences. S3 Table lists for each country of affiliation the first and last names of a random selection of researchers according to their country of origin, as estimated by NamSor. To build this table we used ’listsome’, a Stata module to list a random sample of observations. Finally, S4 and S5 Tables show the first name, last name and country of origin of all researchers with inference accuracy ≥70%, for respectively Japan, a country whose names were well recognized by NamSor, and Kenya, a country whose names were less recognized by NamSor.

All results obtained in the study were similar using the full sample and the subsample consisting only of authors with a single affiliation. As shown in Table 2, the proportion of correct classifications varied widely by country, and was higher for “continent of origin”, compared to “country of origin” and “ethnicity”. Most of the names were correctly identified for some countries, such as Polish, Pakistani and Vietnamese names. Other names were poorly recognized, for example Nepalese or Tanzanian names, and others were not recognized at all by NamSor, mainly Latin American names. No Brazilian, Mexican, Filipino or Cuban names were correctly identified. Brazilian names were mostly considered Portuguese, while Mexican or Cuban names were mostly considered Spanish (S2 Table).

S3 Table shows that NamSor could also be wrong with some names when the first name suggested a different country of origin than the surname. For example, Karol Deutsch is a researcher affiliated with a university in Poland. Although Karol is a common first name in Poland, Namsor identified this researcher as being of German origin, probably because of his surname (’Deutsch’). Similarly, although Erika Marie Bascos’ country of affiliation is the Philippines, Namsor considered this researcher to be of French origin, probably because of her first name (’Erika Marie’). Finally, NamSor was of course mistaken with foreign researchers, or more broadly with researchers with names suggesting a country of origin different from the country of affiliation. For example, Muhammad Bilal and Abdullah Al Mamun were (correctly) identified by NamSor as being of Pakistani and Bangladeshi origin respectively, but these were misclassifications because these researchers are affiliated with Chinese universities.

The use of two modified variables (continent#2 and country#2) increased for all countries the proportion of correct classifications. In addition, by restricting the analyses to subsamples, NamSor’s performance tended to increase gradually as the accuracy threshold value increased. For example, for "country of origin", the proportion of correct classifications for Japan was 85.7% for the full sample (and 86.7% for authors with a single affiliation), 86.3% for a threshold value of 40%, 89.1% for a threshold value of 50%, 90.1% for a threshold value of 60%, and 91.1% for a threshold value of 70%. Similarly, the number of non-classifications also gradually increased as the accuracy threshold value increased. For example, for the same variable (country of origin) and the same country (Japan), the number of names classified by NamSor was 6,362 for the full sample, 6,308 with a cut-off value of 40%, 6,032 with a cut-off value of 50%, 5,905 with a cut-off value of 60%, and 5,732 with a cut-off value of 70%.

As shown in the confusion matrices (Table 3), there was a decrease in the number of correct classifications as the threshold value for inference accuracy increased, due to a greater increase in the number of non-classifications relative to the decrease in the number of misclassifications. For example, for “country of origin”, the number of correct classifications was 64,499 for the full sample, 63,901 with a threshold value of 40%, 58,608 with a threshold value of 50%, 55,149 with a threshold value of 60%, and 50,679 with a threshold value of 70%.

Table 4 (accuracy metrics) confirms the results of the previous table. The proportion of misclassifications and non-classifications (i.e., errorCoded) was lowest for the full sample and for authors with a single affiliation, and increased gradually as the threshold value increased. With a cut-off value of 40%, errorCoded increased only slightly compared to the full sample because the number and proportion of non-classifications (= naCoded) was low: 2,259 (2.6%) for “continent of origin” and “country of origin”, and 6,028 (6.8%) for “ethnicity”. Above 60%, errorCoded reached or exceeded 25% for “continent of origin” and “country of origin” and 15% for “ethnicity”. Using a cut-off value of 50% was probably the strategy that provided the best compromise between “proportion of correct classifications” and “proportion of non-classifications”. For the full sample, the subsample consisting only of authors with a single affiliation, and the subsample with inference accuracy ≥50%, the proportion of misclassifications (= errorCodedWithoutNA) was, respectively, 16.0%, 15.6% and 12.6% for “continent of origin”, 6.3%, 5.7% and 3.3% for “continent#2”, 27.3%, 26.5% and 19.5% for “country of origin”, 19.7%, 18.6% and 11.4% for “country#2”, and 20.2%, 18.8% and 14.8% for “ethnicity”.

As expected, the total proportion of foreign researchers in the study sample can be estimated to be less than 5%, since the proportion of names that were misclassified for "country#2", by including in the analysis only those researchers for whom the country of origin was determined with ≥70% accuracy, was 5.0% (Table 3).

## Discussion

### Main findings

In this cross-sectional study, we examined the performance of NamSor in determining the origin of individuals based on their first and last names. To this end, we used a database of researchers whose country of affiliation was known. We limited the analysis to researchers affiliated with low immigration countries (i.e., <2.5%). We considered the country of origin of these researchers to be their country of affiliation.

All results obtained in the study were similar using the full sample and the subsample consisting only of authors with a single affiliation. We found NamSor to be accurate in determining the continent of origin, especially when using the modified variable (continent#2) and restricting the analysis to names with an inference accuracy ≥50%. For continent#2, the proportion of misclassifications (i.e., errorCodedWNA) was only 6.3% for the full sample, 5.7% for authors with a single affiliation, and 3.3% for the subsample with inference accuracy ≥50%. However, we found that the risk of misclassification was higher with country of origin or ethnicity, but also decreased when using the modified variable (country#2) and the subsample.

### Comparison with existing literature

Several authors used Namsor in the past to estimate the origin of individuals in their research, both in medicine [10, 12] and in other disciplines [21, 22], but our study is the first to our knowledge to have evaluated its performance. We already evaluated NamSor’s performance in determining the gender of individuals from their first and last names, and showed that the tool was accurate in the majority of cases [13]. However, we found that NamSor was much less efficient for some countries, for example for Chinese names [18]. We also found that the use of the accuracy parameter (’probabilityCalibrated’) was not useful to improve the performance of NamSor for gender estimation [23].

The results we obtained in the current study were quite different. Asian names were in general relatively well recognized by NamSor. For example, 76% of the names of researchers affiliated with universities or research institutes in China were correctly classified for “country of origin” (and even 85% for “ethnicity”). These figures were 86% and 85%, respectively, for Japan. The results were similar for authors with a single affiliation (China: 76% for “country of origin” and 84% for “ethnicity”; Japan: 87% for both variables). Furthermore, the use of the accuracy parameter greatly improved the performance of the tool for origin. The best compromise between improving NamSor’s performance and increasing the number of non-classifications was obtained with a threshold value of 50%. With a threshold value of 40%, too few queries were considered as non-classifications (2.6% for “continent of origin” and “country of origin”, and 6.8% for “ethnicity”) to make a noticeable change in performance metrics. For example, for “continent of origin” and “country of origin”, errorcodedWNA decreased only from 16.0% to 15.4% and from 27.3% to 26.1%, while these proportions decreased to 12.6% and 19.5%, respectively, for a threshold value of 50%.

As expected, using “continent of origin” yielded more accurate assignments than either “country of origin” or “ethnicity”. This is a logical finding since “continent of origin” consisted of only three categories, far fewer than the other two variables. For example, if authors with Chinese names were considered to be of Japanese origin, the continent of origin (i.e., Asia) would have been correctly estimated, unlike country of origin or ethnicity. However, if researchers using NamSor needed more precision for their study than simply assigning a continent of origin, the use of “ethnicity” would a priori allow more accurate queries than “country of origin”. For example, for the total sample and for authors with a single affiliation, errorCodedWNA was respectively 20.2% and 18.8% for “ethnicity” and 27.3% and 26.5% for “country of origin”. This difference persisted with the various subsamples.

As expected, it was the joint use of “continent#2” or “country#2” and the various subsamples with threshold values of 50% or more that really improved the performance of NamSor. For “continent#2” and a cut-off value of 50%, the proportion of misclassifications was only 2.6% in our study (vs., for “continent of origin”, 16.0% for the total sample and 15.6% for authors with a single affiliation). For “country#2” and the same cut-off value of 50%, this proportion was 9.3% (vs., for “country of origin”, 27.3% for the total sample and 26.5% for authors with a single affiliation). “Continent#2” led to more accurate assignments than “continent of origin”, as many researchers with Spanish or Portuguese names were actually affiliated with universities or research institutes in Latin America. For the same reason, replacing “country of origin” by “country#2” (i.e., "Spain" by "Spain or Hispanic American country", and "Portugal" by "Portugal or Brazil") was also useful for improving NamSor’s performance.

Anglo-Saxon countries (i.e., UK, USA, Canada, Australia and New Zealand) were excluded from the study, as the proportion of migrants was too high in these countries. However, it is likely that if they were included we would observe misclassifications for the same reason as for names of Spanish or Portuguese origin. It would therefore make sense to use a third variable (country#3) that would add a modification to "country#2", replacing "UK", "USA", "Canada", "Australia" and "New Zealand" with "UK or USA or Canada or Australia or New Zealand".

In recent years, there has been growing recognition of the impact of artificial intelligence and other mechanisms on gender equality and biases across various domains. Three papers by Bao & Huang shed light on this topic. The first paper explored how artificial intelligence (AI) can contribute to creating a gender-neutral learning environment, reducing gender disparities in education [24]. The study compared the results of students in the game of Go with human teachers vs. AI trainers. With human teachers, boys consistently had a higher winning rate than girls, whereas the use of AI trainers led to improvements in the performance of both male and female students. The two other papers highlighted the importance of addressing gender-specific favoritism in scientific recruitment processes, particularly in prestigious scientific committees [25, 26]. Greater female representation in these committees could lead to innovative approaches and managerial effectiveness in shaping research resource allocation and public projects. Bao & Huang discussed the underrepresentation of women in top scientific positions and the need to reform scientific election procedures to foster gender balance, illustrating how gender-specific biases can impact career success and exacerbate gender disparities. These three papers underscore the broader context within which our study of NamSor’s ability to predict individuals’ country of origin and ethnicity takes place. The accurate name-based classification offered by NamSor serves as a critical tool in addressing biases, improving diversity, and promoting equity in research and various decision-making processes.

### Implications for practice and research

The performance of NamSor in determining the origin of individuals was probably underestimated in our study, as it was based on the assumption that all researchers affiliated with universities or research institutes in a given country were from that country. This assumption is not entirely correct, as the countries of affiliation could be included in the study up to a threshold in the proportion of migrants of 2.5%. In addition, researchers are a priori more mobile than the general population and the proportion of foreign researchers is expected to be above the 2.5% threshold for a number of countries. However, this proportion was probably less than 5% in the study since the proportion of misclassification for “country#2”, which includes misclassification related to foreign researchers, was 5.0% using the sample consisting of all names for which the determination was made with at least 70% accuracy.

The fact that the estimate of the performance of NamSor is rather conservative means that using this tool in research following the procedure proposed in the study is probably safe. However, finer determinations of the origin of individuals, at the level of country rather than continent, could possibly also be an option. In order to demonstrate the validity of this strategy, further studies would be needed, which could rely for example on self-identification or the expertise of linguists or onomatologists to assess the performance of NamSor for a large number of countries. Unfortunately, such studies are often difficult to conduct if the number of participants is large, which would be necessary in order to have a wide variety of names represented. Future studies could also be used to compare NamSor to other similar tools that estimate the origin of individuals based on their names, for example NamePrism, a name-based nationality and ethnicity classification, and ethnicolr, a name-based race and ethnicity classification.

NamSor’s potential applications extend to various stakeholders, including researchers, institutions, and policymakers. Researchers can harness the power of NamSor to evaluate and address issues related to discrimination based on individuals’ origin in academic collaborations, funding decisions, and research recognition. By leveraging NamSor’s predictions, research institutions and funding agencies can foster a more diverse and inclusive academic environment. For example, NamSor could facilitate targeted initiatives aimed at addressing underrepresentation of certain groups in research. This includes allocating resources to support underrepresented researchers or encouraging collaborations that bridge diverse backgrounds. The tool can also serve as a valuable resource for ensuring equity in research evaluations, such as grant allocations and award nominations. Furthermore, policymakers can utilize NamSor’s capabilities to inform and shape evidence-based policies that promote diversity, inclusivity, and global collaboration in research. By recognizing and addressing disparities, decision-makers can take steps to enhance the international research landscape. Therefore, NamSor’s role in improving the accuracy of origin predictions has wide-reaching implications, contributing to a more equitable and inclusive research community.

### Limitations

Our study has a large sample size but has two main limitations. First, we restricted the study to twenty-two countries spread over four continents (Europe, Asia, Africa and America). As the performance of NamSor varies depending on the country examined, our results are not necessarily generalizable to other countries. We therefore recommend some changes in the variables used by NamSor. We recommend the use of "continent#2" (i.e., "Europe" replaced by "Europe/America/Oceania") instead of "continent of origin", and the use of "country#3" (i.e., "UK", "USA", "Canada", "Australia" and "New Zealand" replaced in country#2 by "UK or USA or Canada or Australia or New Zealand") instead of "country of origin" or “country#2”. Second, as already stated above, we considered the country of origin of the researchers to be their country of affiliation. Although we restricted the study to countries with less than 2.5% migrants to obtain the most homogeneous populations possible with names representative of the selected countries, there were inevitably foreign researchers in these countries. The results of our study are therefore probably an underestimate of the real performance of NamSor. It would have been better to determine the (actual) origin of the researchers by self-identification, linguistic analysis, or consultation of experts in onomastics. Furthermore, exploring multiple proxies for “country of origin”, combining various sources of information, could offer a more robust approach to ascertain the true origin of individuals. It is essential to recognize that the complexity of individuals’ origin, identity, and mobility necessitates a multifaceted approach to validation.

## Conclusion

NamSor is accurate in determining the continent of origin of individuals from their first and last names, especially when using the modified variable (i.e., continent#2) and restricting the analysis to names with inference accuracy ≥ 50%. The risk of misclassification is higher with country of origin or ethnicity, but decreases, as with continent of origin, when using the modified variable (i.e., country#2) and the subsample. Further research would be useful in the future, as the performance of NamSor was probably underestimated in our study due to the relatively high mobility of researchers. Future investigations could also involve the comparison of NamSor with other name-based classification algorithms, such as NamePrism and ethnicolr, or explore avenues for enhancing the accuracy of NamSor’s predictions through advanced machine learning techniques and more extensive name databases. Such endeavors are essential to advance the understanding of origin determination techniques and their applications across various fields.

### Preprint

A preprint of this manuscript was posted on Research Square [27].

## Supporting information

S1 Table Python program to retrieve all PubMed articles published in 2021 with at least one author affiliated with a university or research institute in China (adapted from https://github.com/gijswobben/pymed). The same procedure was followed for the other countries of affiliation.
(DOCX)

S2 Table Number and proportion of researchers by country of origin of researchers (five countries of origin, ranked by the number of inferences, are shown for each country of affiliation). Data are presented for the full sample and for two subsamples including only names for which the accuracy of inference was, respectively, ≥50% and ≥70%.
(DOCX)

S3 Table First and last names of a random selection of researchers (sorted by country of affiliation and country of origin). (DOCX)

S4 Table First name, last name and country of origin of all researchers with inference accuracy ≥70% for a country whose names were well recognized by NamSor (i.e., Japan). (XLS)

S5 Table First name, last name and country of origin of all researchers with inference accuracy ≥70% for a country whose names were relatively poorly recognized by NamSor (i.e., Kenya). (XLS)
