# Supplementary material for: How well does NamSor perform in predicting the country of origin and ethnicity of individuals based on their first and last names?
Source: PLoS One. 2023 Nov 16;18(11):e0294562. doi: 10.1371/journal.pone.0294562 (PMC10653483; doi:10.1371/journal.pone.0294562)
Supplement: S1 Table — The same procedure was followed for the other countries of affiliation. (DOCX) [file pone.0294562.s001.docx]

S1 Table. Python program to retrieve all PubMed articles published in 2021 with at least one author affiliated with a university or research institute in China (adapted from https://github.com/gijswobben/pymed). The same procedure was followed for the other countries of affiliation.

from pymed import PubMed

import pandas as pd

import numpy as np

import csv as df

pubmed = PubMed(tool="PubMedSearcher", email="paulsebo@hotmail.com")

## PUT YOUR SEARCH TERM HERE ##

search_term = '((China[affiliation])) AND ((“2021/01/01”[edat]:”2021/12/31”[edat]))'

results = pubmed.query(search_term, max_results=1000000)

articleList = []

articleInfo = []

for article in results:

# Print the type of object we've found.

# We need to convert it to dictionary with available function

articleDict = article.toDict()

articleList.append(articleDict)

# Generate list of dict records which will hold all article details that could be fetch from PUBMED API

for article in articleList:

#Sometimes article['pubmed_id'] contains list separated with comma - take first pubmedId in that list - thats article pubmedId

pubmedId = article['pubmed_id'].partition('\n')[0]

# Append article info to dictionary

articleInfo.append({u'pubmed_id':pubmedId,

u'publication_date':article['publication_date'],

u'authors':article['authors']})

# Generate Pandas DataFrame from list of dictionaries

articlesPD = pd.DataFrame.from_dict(articleInfo)

df = articlesPD

export_csv = df.to_csv (r'C:\Users\seboe\Documents\excel_csv_txt\export_pymed_origin_China.csv', index = None, header=True)

#Print first 10 rows of dataframe

print(articlesPD.head(10))
