# Supplementary material for: How well does NamSor perform in predicting the country of origin and ethnicity of individuals based on their first and last names?
Source: PLoS One. 2023 Nov 16;18(11):e0294562. doi: 10.1371/journal.pone.0294562 (PMC10653483; doi:10.1371/journal.pone.0294562)
Supplement: S2 Table — Data are presented for the full sample and for two subsamples including only names for which the accuracy of inference was, respectively, ≥50% and ≥70%. (DOCX) [file pone.0294562.s002.docx]

S2 Table. Number and proportion of researchers by country of origin of researchers (five countries of origin, ranked by the number of inferences, are shown for each country of affiliation). Data are presented for the full sample and for two subsamples including only names for which the accuracy of inference was, respectively, ≥50% and ≥70%.

| Country of affiliation of researchers (countries ranked by number of medical publications in 2020) | Country of origin of researchers (estimated by NamSor), Full sample | N (%) | Country of origin of researchers (estimated by NamSor), Accuracy ≥50% | N (%) | Country of origin of researchers (estimated by NamSor), Accuracy ≥70% | N (%) |
| --- | --- | --- | --- | --- | --- | --- |
| China |  |  |  |  |  |  |
|  | China | 5837 (75.8) | China | 4862 (80.4) | China | 3646 (80.1) |
|  | Pakistan | 741 (9.6) | Pakistan | 693 (11.5) | Pakistan | 618 (13.6) |
|  | Taiwan | 567 (7.4) | Taiwan | 161 (2.7) | Taiwan | 54 (1.2) |
|  | Hong Kong | 94 (1.2) | Hong Kong | 46 (0.8) | Ghana | 38 (0.8) |
|  | Bangladesh | 55 (0.7) | Ghana | 42 (0.7) | Bangladesh, Hong Kong | 28 (0.6) |
| Japan |  |  |  |  |  |  |
|  | Japan | 5451 (85.7) | Japan | 5374 (89.1) | Japan | 5223 (91.1) |
|  | China | 208 (3.3) | China | 173 (2.9) | China | 137 (2.4) |
|  | Bangladesh | 67 (1.1) | Bangladesh | 54 (0.9) | Viet Nam | 45 (0.8) |
|  | Indonesia | 52 (0.8) | Viet Nam | 46 (0.8) | Bangladesh | 42 (0.7) |
|  | Viet Nam | 48 (0.8) | Indonesia, South Korea | 37 (0.6) | South Korea | 35 (0.6) |
| India |  |  |  |  |  |  |
|  | India | 3406 (63.5) | India | 2530 (75.1) | India | 1521 (79.4) |
|  | Bangladesh | 379 (7.1) | Pakistan | 235 (7.0) | Pakistan | 176 (9.2) |
|  | Sri Lanka | 367 (6.8) | Bangladesh | 202 (6.0) | Bangladesh | 105 (5.5) |
|  | Pakistan | 345 (6.4) | Sri Lanka | 136 (4.0) | Sri Lanka | 34 (1.8) |
|  | Mauritius | 221 (4.1) | Mauritius | 62 (1.8) | Mauritius | 12 (0.6) |
| Brazil |  |  |  |  |  |  |
|  | Portugal | 1635 (57.8) | Portugal | 1429 (68.1) | Portugal | 1162 (76.5) |
|  | Italy | 367 (13.0) | Italy | 285 (13.6) | Italy | 176 (11.6) |
|  | Spain | 339 (12.0) | Spain | 190 (9.1) | Spain | 109 (7.2) |
|  | France | 92 (3.3) | France | 40 (1.9) | France | 14 (0.9) |
|  | Germany | 76 (2.7) | Germany | 40 (1.9) | Germany | 13 (0.9) |
| Poland |  |  |  |  |  |  |
|  | Poland | 16816 (91.2) | Poland | 15814 (95.2) | Poland | 14313 (97.0) |
|  | Slovakia | 319 (1.7) | Ukraine | 104 (0.6) | Ukraine | 75 (0.5) |
|  | Germany | 169 (0.9) | Slovakia | 74 (0.5) | Italy | 40 (0.3) |
|  | Ukraine | 126 (0.7) | Germany | 68 (0.4) | Iran | 33 (0.2) |
|  | Czech Republic | 78 (0.4) | Italy | 55 (0.3) | Spain | 29 (0.2) |
| Egypt |  |  |  |  |  |  |
|  | Egypt | 8615 (90.9) | Egypt | 7889 (96.9) | Egypt | 6553 (98.8) |
|  | Saudi Arabia | 266 (2.8) | Saudi Arabia | 97 (1.2) | Saudi Arabia | 37 (0.6) |
|  | Pakistan | 84 (0.9) | Pakistan | 33 (0.4) | Pakistan | 9 (0.1) |
|  | Morocco | 60 (0.6) | Morocco | 13 (0.2) | France, | 3 (0.1) |
|  | Algeria | 54 (0.6) | Syria | 12 (0.2) | Ireland, Malaysia, Tunisia | 3 (0.1) |
| Mexico |  |  |  |  |  |  |
|  | Spain | 4845 (82.6) | Spain | 4199 (90.7) | Spain | 3295 (94.8) |
|  | Portugal | 175 (3.0) | Italy | 84 (1.8) | Italy | 43 (1.2) |
|  | Italy | 173 (3.0) | France | 65 (1.4) | France | 23 (0.7) |
|  | France | 109 (1.9) | Portugal | 57 (1.2) | Portugal | 17 (0.5) |
|  | Romania | 58 (1.0) | Ireland | 30 (0.7) | Ireland, India | 11 (0.3) |
| Pakistan |  |  |  |  |  |  |
|  | Pakistan | 6388 (93.8) | Pakistan | 6035 (97.3) | Pakistan | 5340 (98.8) |
|  | Bangladesh | 110 (1.6) | Bangladesh | 45 (0.7) | Bangladesh | 18 (0.3) |
|  | India | 41 (0.6) | India | 28 (0.5) | India | 15 (0.3) |
|  | Malaysia | 33 (0.5) | Malaysia | 13 (0.2) | Iran | 8 (0.2) |
|  | Egypt | 31 (0.5) | Egypt | 12 (0.2) | Egypt | 6 (0.1) |
| Indonesia |  |  |  |  |  |  |
|  | Indonesia | 2980 (77.9) | Indonesia | 2644 (87.6) | Indonesia | 2291 (93.5) |
|  | Malaysia | 178 (4.7) | Malaysia | 104 (3.5) | Pakistan | 53 (2.2) |
|  | Pakistan | 128 (3.3) | Pakistan | 78 (2.6) | Malaysia | 37 (1.5) |
|  | Italy | 52 (1.4) | Italy | 23 (0.8) | Italy | 9 (0.4) |
|  | Ireland | 45 (1.2) | Ireland | 17 (0.6) | Japan | 9 (0.4) |
| Nigeria |  |  |  |  |  |  |
|  | Nigeria | 2553 (75.8) | Nigeria | 2352 (87.3) | Nigeria | 2129 (93.7) |
|  | Ghana | 139 (4.1) | Ghana | 61 (2.3) | Pakistan | 35 (1.5) |
|  | Kenya | 107 (3.2) | Pakistan | 56 (2.1) | Ghana | 25 (1.1) |
|  | Pakistan | 94 (2.8) | Kenya | 40 (1.5) | Kenya | 18 (0.8) |
|  | Niger | 68 (2.0) | Niger | 38 (1.4) | Niger | 14 (0.6) |
| Iraq |  |  |  |  |  |  |
|  | Iraq | 270 (26.8) | Iraq | 171 (33.7) | Iraq | 95 (42.2) |
|  | Saudi Arabia | 215 (21.4) | Pakistan | 108 (21.3) | Saudi Arabia | 43 (19.1) |
|  | Pakistan | 193 (19.2) | Saudi Arabia | 98 (19.3) | Pakistan | 40 (17.8) |
|  | Egypt | 123 (12.2) | Egypt | 57 (11.2) | Egypt | 26 (11.6) |
|  | Syria | 49 (4.9) | Syria | 18 (3.6) | Syria | 5 (2.2) |
| Ethiopia |  |  |  |  |  |  |
|  | Ethiopia | 3671 (91.1) | Ethiopia | 3556 (96.5) | Ethiopia | 3448 (98.8) |
|  | Egypt | 36 (0.9) | Egypt | 16 (0.4) | Niger | 7 (0.2) |
|  | Pakistan | 26 (0.7) | Pakistan | 16 (0.4) | Switzerland | 4 (0.1) |
|  | Niger | 25 (0.6) | Niger | 11 (0.3) | Finland | 3 (0.1) |
|  | Namibia, Saudi Arabia | 18 (0.5) | Saudi Arabia | 9 (0.2) | Kenya, Pakistan, South Korea | 3 (0.1) |
| Bangladesh |  |  |  |  |  |  |
|  | Bangladesh | 1955 (78.5) | Bangladesh | 1784 (86.9) | Bangladesh | 1565 (93.9) |
|  | Pakistan | 307 (12.3) | Pakistan | 192 (9.4) | Pakistan | 75 (4.5) |
|  | India | 64 (2.6) | India | 34 (1.7) | India | 15 (0.9) |
|  | Egypt | 22 (0.9) | Egypt | 8 (0.4) | Egypt | 3 (0.2) |
|  | Saudi Arabia | 19 (0.8) | Syria | 6 (0.3) | Saudi Arabia | 3 (0.2) |
| Viet Nam |  |  |  |  |  |  |
|  | Viet Nam | 1842 (94.0) | Viet Nam | 1837 (95.5) | Viet Nam | 1828 (96.8) |
|  | Iran | 19 (1.0) | Iran | 19 (1.0) | Iran | 15 (0.8) |
|  | Ireland | 10 (0.5) | Ireland | 8 (0.4) | Nigeria | 6 (0.3) |
|  | India | 7 (0.4) | India | 6 (0.3) | Pakistan | 5 (0.3) |
|  | Netherlands | 7 (0.4) | Nigeria, Pakistan | 6 (0.3) | India, Ireland | 4 (0.2) |
| Tunisia |  |  |  |  |  |  |
|  | Tunisia | 1224 (75.0) | Tunisia | 999 (90.6) | Tunisia | 684 (95.0) |
|  | Algeria | 149 (9.1) | Algeria | 55 (5.0) | Algeria | 14 (1.9) |
|  | Morocco | 133 (8.2) | Morocco | 19 (1.7) | Morocco | 7 (1.0) |
|  | Egypt | 37 (2.3) | Egypt | 9 (0.8) | Burkina Faso | 5 (0.7) |
|  | Niger, Saudi Arabia | 14 (0.9) | Burkina Faso | 5 (0.5) | Egypt | 4 (0.6) |
| Kenya |  |  |  |  |  |  |
|  | Kenya | 665 (56.0) | Kenya | 582 (69.7) | Kenya | 489 (77.7) |
|  | Nigeria | 81 (6.8) | Ireland | 36 (4.3) | Nigeria | 19 (3.0) |
|  | Ireland | 57 (4.8) | Nigeria | 21 (2.5) | Ethiopia | 16 (2.5) |
|  | Congo | 43 (3.6) | Ethiopia | 18 (2.2) | Ireland | 13 (2.1) |
|  | Tanzania | 28 (2.4) | India, Pakistan | 14 (1.7) | Tanzania | 8 (1.3) |
| Morocco |  |  |  |  |  |  |
|  | Morocco | 1091 (70.6) | Morocco | 789 (84.5) | Morocco | 507 (89.0) |
|  | Algeria | 180 (11.7) | Algeria | 40 (4.3) | Niger | 20 (3.5) |
|  | Niger | 49 (3.2) | Niger | 29 (3.1) | Algeria | 7 (1.2) |
|  | Tunisia | 47 (3.0) | Egypt | 12 (1.3) | Mauritania | 6 (1.1) |
|  | Egypt | 41 (2.7) | Tunisia | 9 (1.0) | Egypt, Rwanda | 5 (0.9) |
| Nepal |  |  |  |  |  |  |
|  | India | 476 (35.9) | India | 263 (40.2) | India | 131 (48.3) |
|  | Nepal | 406 (30.6) | Nepal | 233 (35.6) | Nepal | 87 (32.1) |
|  | Sri Lanka | 113 (8.5) | Sri Lanka | 33 (5.0) | Pakistan | 14 (5.2) |
|  | Bangladesh | 82 (6.2) | Bangladesh | 31 (4.7) | Bangladesh | 8 (3.0) |
|  | Indonesia | 55 (4.1) | Indonesia | 27 (4.1) | Ireland, Sri Lanka | 7 (2.6) |
| Ghana |  |  |  |  |  |  |
|  | Ghana | 1036 (74.9) | Ghana | 945 (86.1) | Ghana | 839 (91.5) |
|  | Ireland | 36 (2.6) | Nigeria | 22 (2.0) | Nigeria | 18 (2.0) |
|  | Kenya | 29 (2.1) | Ireland | 16 (1.5) | Ireland | 6 (0.7) |
|  | Nigeria | 25 (1.8) | Kenya | 16 (1.5) | Kenya | 6 (0.7) |
|  | Cameroon | 20 (1.5) | Benin | 10 (0.9) | Liberia | 5 (0.6) |
| Philippines |  |  |  |  |  |  |
|  | Spain | 380 (34.1) | Spain | 210 (41.2) | Spain | 119 (52.7) |
|  | France | 140 (12.6) | France | 55 (10.8) | France | 17 (7.5) |
|  | Ireland | 110 (9.9) | Ireland | 50 (9.8) | Ireland | 15 (6.6) |
|  | UK | 45 (4.0) | UK | 26 (5.1) | China | 8 (3.5) |
|  | Germany | 31 (2.8) | India | 19 (3.7) | India | 8 (3.5) |
| Tanzania |  |  |  |  |  |  |
|  | Tanzania | 293 (43.5) | Tanzania | 212 (54.8) | Tanzania | 121 (57.4) |
|  | Kenya | 72 (10.7) | Kenya | 27 (7.0) | Kenya | 17 (8.1) |
|  | Congo | 41 (6.1) | Pakistan | 16 (4.1) | Pakistan | 12 (5.7) |
|  | Zimbabwe | 27 (4.0) | Congo | 14 (3.6) | Italy | 7 (3.3) |
|  | Pakistan | 25 (3.7) | Zimbabwe | 14 (3.6) | India, Nigeria, Rwanda | 5 (2.4) |
| Cuba |  |  |  |  |  |  |
|  | Spain | 261 (85.6) | Spain | 225 (94.9) | Spain | 184 (97.9) |
|  | France | 9 (3.0) | France | 2 (0.8) | China | 1 (0.5) |
|  | Portugal | 6 (2.0) | Ireland | 2 (0.8) | France | 1 (0.5) |
|  | Italy | 4 (1.3) | Portugal | 2 (0.8) | Ireland | 1 (0.5) |
|  | Netherlands | 4 (1.3) | Russia | 2 (0.8) | Portugal | 1 (0.5) |
