# Supplementary material for: How well does NamSor perform in predicting the country of origin and ethnicity of individuals based on their first and last names?
Source: PLoS One. 2023 Nov 16;18(11):e0294562. doi: 10.1371/journal.pone.0294562 (PMC10653483; doi:10.1371/journal.pone.0294562)
Supplement: S3 Table — (DOCX) [file pone.0294562.s003.docx]

S3 Table. First and last names of a random selection of researchers (sorted by country of affiliation and country of origin).

| Country of affiliation of researchers (countries ranked by number of medical publications in 2020) | Country of origin of researchers (estimated by NamSor) | N (%) | Random selection of researchers' names, sorted by country of affiliation and country of origin (first names) | Random selection of researchers' names, sorted by country of affiliation and country of origin (last names) | Inference accuracy for country of origin (min=0, max=1) |
| --- | --- | --- | --- | --- | --- |
| China |  |  |  |  |  |
|  | China | 5837 (75.8) | Weilong | Li | 0.45 |
|  |  |  | Hongying | Li | 0.43 |
|  |  |  | Liang | Liang | 0.46 |
|  |  |  | Sijun | Liu | 0.77 |
|  |  |  | Peng | Liu | 0.47 |
|  |  |  | Jian | Lu | 0.65 |
|  |  |  | Yan-Ping | Wang | 0.47 |
|  |  |  | Haijun | Wang | 0.83 |
|  |  |  | Weilin | Xu | 0.85 |
|  |  |  | Ke-Da | Yu | 0.36 |
|  | Pakistan | 741 (9.6) | Muhammad | Bilal | 0.90 |
|  |  |  | Abbas Ali | Chandio | 0.70 |
|  |  |  | Muhammad Hammad | Hussain | 0.92 |
|  |  |  | Ishtiaq | Ahmed | 0.66 |
|  |  |  | Temoor | Ahmed | 0.84 |
|  |  |  | Imran Ullah | Shah | 0.98 |
|  |  |  | Assar Ali | Shah | 0.94 |
|  |  |  | Shafeeq | Ur Rahman | 0.44 |
|  |  |  | Muhammad Azeem | Ashraf | 0.99 |
|  |  |  | Madiha | Zaynab | 0.47 |
|  | Taiwan | 567 (7.4) | Li | Chen | 0.54 |
|  |  |  | Jingxuan | Chen | 0.38 |
|  |  |  | Yang | Cheng | 0.42 |
|  |  |  | Yun-Wu | Li | 0.49 |
|  |  |  | Yi-Chen | Li | 0.61 |
|  |  |  | Shen | Li | 0.43 |
|  |  |  | Fang-Ru | Li | 0.58 |
|  |  |  | Mingchen | Liu | 0.42 |
|  |  |  | Yu | Liu | 0.34 |
|  |  |  | Ting | Lu | 0.42 |
|  | Hong Kong | 94 (1.2) | Adrian Chi Heng | Fung | 0.93 |
|  |  |  | Yiming | Kong | 0.46 |
|  |  |  | Kai | Li | 0.56 |
|  |  |  | Kai | Liu | 0.46 |
|  |  |  | Kai | Lu | 0.54 |
|  |  |  | Kai | Ma | 0.60 |
|  |  |  | Sai-Ying | Wan | 0.42 |
|  |  |  | Andy Wai Kan | Yeung | 0.99 |
|  |  |  | Ping | Yi | 0.42 |
|  |  |  | Cho-Tung | Yip | 0.92 |
|  | Bangladesh | 55 (0.7) | Md Mahadi | Hasan | 0.91 |
|  |  |  | Najmul | Hasan | 0.88 |
|  |  |  | Yasir | Islam | 0.46 |
|  |  |  | Hasan | Mahmud | 0.60 |
|  |  |  | Abdullah Al | Mamun | 0.74 |
|  |  |  | Easar | Alam | 0.68 |
|  |  |  | Md Kaisar | Ali | 0.84 |
|  |  |  | Mohammed | Shakib | 0.43 |
|  |  |  | Md Salman | Sohel | 0.99 |
|  |  |  | Mohammed Sharif | Swallah | 0.25 |
| Japan |  |  |  |  |  |
|  | Japan | 5451 (85.7) | Shunsuke | Hanaki | 0.99 |
|  |  |  | Tadaaki | Hanatani | 0.92 |
|  |  |  | Takahiro | Ikoma | 0.98 |
|  |  |  | Satoshi | Katayama | 0.97 |
|  |  |  | Yushi | Katsuyama | 0.95 |
|  |  |  | Shinpei | Matsuda | 0.93 |
|  |  |  | Yukiko | Nakano | 0.99 |
|  |  |  | Tetsuji | Noguchi | 0.99 |
|  |  |  | Hitomi | Takei | 0.99 |
|  |  |  | Yoichi | Toyoshima | 0.99 |
|  | China | 208 (3.3) | Jing | Che | 0.55 |
|  |  |  | Zhongyuan | Feng | 0.90 |
|  |  |  | Qing | Liu | 0.89 |
|  |  |  | Yuxiong | Lu | 0.76 |
|  |  |  | Xiaowen | Wang | 0.86 |
|  |  |  | Shenghan | Gao | 0.83 |
|  |  |  | Hainan | Yue | 0.79 |
|  |  |  | Yunliang | Zang | 0.92 |
|  |  |  | Lulu | Zhang | 0.91 |
|  |  |  | Jianhong | Zhu | 0.94 |
|  | Bangladesh | 67 (1.1) | Lata | Chouhan | 0.43 |
|  |  |  | Mohammad Omar | Faruk | 0.42 |
|  |  |  | Muhammad Nazmul | Haque | 0.74 |
|  |  |  | Sifat | Ahmed | 0.61 |
|  |  |  | Sakirul | Khan | 0.65 |
|  |  |  | Rahman Md | Moshikur | 0.74 |
|  |  |  | Md Kawsar | Mustofa | 0.90 |
|  |  |  | Monira | Pervin | 0.83 |
|  |  |  | Mohammad Habibur Rahman | Sarker | 0.99 |
|  |  |  | Nahin Islam | Shiblee | 0.99 |
|  | Indonesia | 52 (0.8) | Harry Handoko | Halim | 0.92 |
|  |  |  | Angga | Hermawan | 0.92 |
|  |  |  | Prihardi | Kahar | 0.61 |
|  |  |  | Nuri | Luthfiana | 0.88 |
|  |  |  | Bangun Satrio | Nugroho | 0.99 |
|  |  |  | Safira Latifa Erlangga | Putri | 0.92 |
|  |  |  | Natasya Trivena | Rokot | 0.66 |
|  |  |  | Ferry Ferdiansyah | Sofian | 0.89 |
|  |  |  | Keeya | Sunata | 0.61 |
|  |  |  | Mina | Waraya | 0.34 |
|  | Taiwan | 47 (0.7) | Sanmei | Chen | 0.38 |
|  |  |  | Chia-Hsiu | Chen | 0.94 |
|  |  |  | Lin-Qing | Chen | 0.46 |
|  |  |  | Hung-Hsin | Huang | 0.94 |
|  |  |  | Yanhang | Chen | 0.42 |
|  |  |  | Yu-Lin | Li | 0.56 |
|  |  |  | Hua | Lin | 0.42 |
|  |  |  | Chen | Liu | 0.48 |
|  |  |  | Yeong Huei | Ng | 0.73 |
|  |  |  | Ji-Wen | Wu | 0.42 |
| India |  |  |  |  |  |
|  | India | 3406 (63.5) | Abhishek | Gangopadhyay | 0.86 |
|  |  |  | Ankita | Aggarwal | 0.90 |
|  |  |  | Kshiteeja | Jain | 0.87 |
|  |  |  | Santosh | Kumar | 0.88 |
|  |  |  | Aditya | Kumar | 0.74 |
|  |  |  | Prasenjit | Mitra | 0.72 |
|  |  |  | Ravindra Attur | Prabhu | 0.41 |
|  |  |  | Bhanudeep | Singanamalla | 0.64 |
|  |  |  | Chandrashekhar | Singh | 0.92 |
|  |  |  | Kavalipurapu Venkata | Teja | 0.78 |
|  | Bangladesh | 379 (7.1) | Sugata Narayan | Biswas | 0.90 |
|  |  |  | Chiranjib | Chakraborty | 0.46 |
|  |  |  | Rimlee | Dutta | 0.42 |
|  |  |  | Tanzeembanu | Gajbar | 0.43 |
|  |  |  | Sahanaz Praveen | Ahmed | 0.43 |
|  |  |  | Monika | Jain | 0.40 |
|  |  |  | Swayansu Sabyasachi | Mohanty | 0.42 |
|  |  |  | Dinabandhu | Mondal | 0.42 |
|  |  |  | Sheikh | Shoib | 0.66 |
|  |  |  | Thirunavukkarasu | Arun Babu | 0.52 |
|  | Sri Lanka | 367 (6.8) | Nishtha | Chawla | 0.48 |
|  |  |  | Sanjith | Aaron | 0.60 |
|  |  |  | Nirupama | Kasturi | 0.53 |
|  |  |  | Usha | Khemani | 0.27 |
|  |  |  | Samir | Mohindra | 0.42 |
|  |  |  | Chavan | Sakshi | 0.50 |
|  |  |  | Rajendra Prasad | Anne | 0.60 |
|  |  |  | Yashaswi | Thummala | 0.42 |
|  |  |  | Jayavardini | Vasanthan | 0.35 |
|  |  |  | Priyanka | Zinge | 0.74 |
|  | Pakistan | 345 (6.4) | Ejaz | Dar | 0.94 |
|  |  |  | Mahak | Fatima | 0.57 |
|  |  |  | Adiba | Khan | 0.55 |
|  |  |  | Farheen | Akhtar | 0.90 |
|  |  |  | Manish | Kumar Mehra | 0.42 |
|  |  |  | Durbar | Maji | 0.50 |
|  |  |  | Arif | Mohammad | 0.55 |
|  |  |  | Romaan | Nazir | 0.90 |
|  |  |  | Imran | Ansari | 0.82 |
|  |  |  | Mir Mahmood | Asrar | 0.87 |
|  | Mauritius | 221 (4.1) | Rajiv | Borah | 0.68 |
|  |  |  | Devansh | Goyal | 0.41 |
|  |  |  | Rakesh | Lodha | 0.46 |
|  |  |  | Varsha | Murthy | 0.44 |
|  |  |  | Vimal | Rajput | 0.47 |
|  |  |  | Jairam | Ramesh | 0.44 |
|  |  |  | Rajendra Kumar | Sahoo | 0.43 |
|  |  |  | Venkatesh | Anehosur | 0.42 |
|  |  |  | Neha | Tavker | 0.42 |
|  |  |  | Ushashi | Banerjee | 0.50 |
| Brazil |  |  |  |  |  |
|  | Portugal | 1635 (57.8) | Kamila Sabino | Batista | 0.68 |
|  |  |  | Gustavo | Cunha de Araújo | 0.99 |
|  |  |  | João Rafael Silva Simões | Estrela | 0.99 |
|  |  |  | Eloiza K | Ferreira | 0.67 |
|  |  |  | Millena Christie | Ferreira Avelar | 0.42 |
|  |  |  | Leticia Alves | Freitas | 0.95 |
|  |  |  | Clarice Paiva | Santana | 0.80 |
|  |  |  | Juliana Akamine | Torrecilhas | 0.42 |
|  |  |  | Thiago | Torres | 0.42 |
|  |  |  | Tatiane Piedade | de Souza | 0.40 |
|  | Italy | 367 (13.0) | Caroline Faria | Bellani | 0.80 |
|  |  |  | Thalita | Galassi | 0.42 |
|  |  |  | Cleide | Garbelini Lima Forneck | 0.42 |
|  |  |  | Glaucia | Pantano | 0.75 |
|  |  |  | Angelica Amorim | Amato | 0.43 |
|  |  |  | Débora | Rebechi | 0.64 |
|  |  |  | Cácia | Signori | 0.84 |
|  |  |  | Desyrre | Tedeschi | 0.77 |
|  |  |  | Alessandro | Urzedo | 0.42 |
|  |  |  | Caio | Zanetti | 0.99 |
|  | Spain | 339 (12.0) | Pablo Cesar Lustosa Barros | Bezerra | 0.68 |
|  |  |  | Anderson Carlos | Camargo | 0.74 |
|  |  |  | Rafael | Corrêa | 0.88 |
|  |  |  | Lorena | Goulart | 0.48 |
|  |  |  | Marcos | Mariano | 0.47 |
|  |  |  | Carlos | Moreira-Neto | 0.36 |
|  |  |  | Claudia | Pidal | 0.43 |
|  |  |  | Ed Carlos | Rey Moura | 0.77 |
|  |  |  | José Leandro | Andrade-Santos | 0.43 |
|  |  |  | Yuliana | Vega-Chacón | 0.71 |
|  | France | 92 (3.3) | Thaís | Barbin | 0.68 |
|  |  |  | Marlene | Benchimol | 0.84 |
|  |  |  | Daniel | Girardi | 0.43 |
|  |  |  | Lissa | Goulart | 0.43 |
|  |  |  | Nicole Soares | Guidony | 0.60 |
|  |  |  | Marilaine | Martins | 0.42 |
|  |  |  | Magáli | Mocellin | 0.69 |
|  |  |  | Aline | Rodrigues | 0.42 |
|  |  |  | Francine | Rubião | 0.42 |
|  |  |  | Christian | Sávio-Silva | 0.42 |
|  | Germany | 76 (2.7) | Angelica Beate Winter | Boldt | 0.95 |
|  |  |  | Heiter | Boness | 0.73 |
|  |  |  | Otto Hernandez | Fustes | 0.45 |
|  |  |  | Juliana Lopes | Hoehne | 0.42 |
|  |  |  | Alvair | Hoffmann | 0.46 |
|  |  |  | Morgana G Martins | Krieger | 0.76 |
|  |  |  | Willian | Lazarini-Lopes | 0.42 |
|  |  |  | Gabriela | Reichert | 0.43 |
|  |  |  | Leonardo | Roever | 0.53 |
|  |  |  | Simone Nardin | Weis | 0.43 |
| Poland |  |  |  |  |  |
|  | Poland | 16816 (91.2) | Agnieszka | Galanty | 0.96 |
|  |  |  | Paweł | Jałoszyński | 0.99 |
|  |  |  | Michal | Kowara | 0.79 |
|  |  |  | Sylwia | Kołtan | 0.99 |
|  |  |  | Roman | Kujawa | 0.53 |
|  |  |  | Paulina | Kęska | 0.78 |
|  |  |  | Anna Maria | Lopatkiewicz | 0.96 |
|  |  |  | Michał | Sabat | 0.96 |
|  |  |  | Katarzyna | Wachowska | 0.99 |
|  |  |  | Karolina | Wojtowicz | 0.99 |
|  | Slovakia | 319 (1.7) | Anna | Baran | 0.43 |
|  |  |  | Helena | Bulińska-Stangrecka | 0.72 |
|  |  |  | Bożena | Bądzyńska | 0.42 |
|  |  |  | Irena | Duś-Ilnicka | 0.42 |
|  |  |  | Ernest | Kuchar | 0.54 |
|  |  |  | Olga | Kuczkiewicz-Siemion | 0.44 |
|  |  |  | Emil | Paluch | 0.42 |
|  |  |  | Renata | Sokolik | 0.37 |
|  |  |  | Józef | Stala | 0.84 |
|  |  |  | Anna | Sęk-Mastej | 0.43 |
|  | Germany | 169 (0.9) | Ralf | Brisch | 0.88 |
|  |  |  | Karol | Deutsch | 0.47 |
|  |  |  | Anna | Egbert | 0.48 |
|  |  |  | Sebastian | Frankowski | 0.42 |
|  |  |  | Beniamin Oskar | Grabarek | 0.91 |
|  |  |  | Anna | Hogendorf | 0.42 |
|  |  |  | Aleksandra | Kroemeke | 0.48 |
|  |  |  | Roland | Pawliczek | 0.34 |
|  |  |  | Olgierd | Stieler | 0.48 |
|  |  |  | August | Wrotek | 0.77 |
|  | Ukraine | 126 (0.7) | Bogdan | Batko | 0.54 |
|  |  |  | Aleksandra | Benko | 0.45 |
|  |  |  | Iuliia | Iavorska | 0.89 |
|  |  |  | Oleksandr | Oliynyk | 0.99 |
|  |  |  | Svitlana | Pastukh | 0.94 |
|  |  |  | Yuriy | Povstenko | 0.87 |
|  |  |  | Ilona | Tomczyk-Wydrych | 0.42 |
|  |  |  | Yuliya | Toporivska | 0.74 |
|  |  |  | Nazar | Trotsko | 0.98 |
|  |  |  | Vitaliy | Yakovyna | 0.86 |
|  | Czech Republic | 78 (0.4) | Kamil | Bechta | 0.39 |
|  |  |  | Anna | Fojt | 0.44 |
|  |  |  | Roman | Jaksik | 0.42 |
|  |  |  | Roman | Kotas | 0.46 |
|  |  |  | Sabina | Krupa | 0.42 |
|  |  |  | Roman | Królik | 0.43 |
|  |  |  | Jakub | Rech | 0.42 |
|  |  |  | Filip | Sadura | 0.65 |
|  |  |  | Nikola | Tułowiecka | 0.43 |
|  |  |  | Nicol | Zielinska | 0.42 |
| Egypt |  |  |  |  |  |
|  | Egypt | 8615 (90.9) | Hend H | Abdullah | 0.80 |
|  |  |  | Amr Ahmed | El-Arabey | 0.99 |
|  |  |  | Fatma | El-Demerdash | 0.94 |
|  |  |  | Waleed | El-Refaie | 0.66 |
|  |  |  | Amr Abd | El-Wahab | 0.99 |
|  |  |  | Sanaa | Ghoneam | 0.80 |
|  |  |  | Magdy Mohamed | Allam | 0.99 |
|  |  |  | Ahmed | Amer | 0.78 |
|  |  |  | Shereen | Sayed | 0.84 |
|  |  |  | Mohamed | Shawky | 0.93 |
|  | Saudi Arabia | 266 (2.8) | Abotalib | Abotalib | 0.59 |
|  |  |  | Mohammed | Elbediwi | 0.42 |
|  |  |  | Abdallah | Ghazalah | 0.46 |
|  |  |  | Aya | Al-Naseri | 0.42 |
|  |  |  | Omkolsoum | Alhaddad | 0.58 |
|  |  |  | Mohamed | Ali | 0.44 |
|  |  |  | Bedeir | Ali-El-Dein | 0.36 |
|  |  |  | Othman | Omar | 0.48 |
|  |  |  | Salah | Aly | 0.42 |
|  |  |  | Taghreed | Shalabi | 0.43 |
|  | Pakistan | 84 (0.9) | Rana | A Youness | 0.54 |
|  |  |  | Mustafa | Abdul Salam | 0.43 |
|  |  |  | Said | Hassan | 0.37 |
|  |  |  | Abdelmonim Ali | Ahmad | 0.47 |
|  |  |  | Gellan | Ahmed | 0.42 |
|  |  |  | Ahmad | Mirza | 0.72 |
|  |  |  | Shereen | Ali | 0.48 |
|  |  |  | Mai | Anwar | 0.47 |
|  |  |  | Aesha | Siddiqui | 0.89 |
|  |  |  | Nasra | Ayuob | 0.51 |
|  | Morocco | 60 (0.6) | Adnan | Bekhit | 0.42 |
|  |  |  | Said | Daboor | 0.29 |
|  |  |  | Hala | El Hanbuli | 0.42 |
|  |  |  | Souad Youssouf Kani | Elmi | 0.51 |
|  |  |  | Abdelgawad | Fahmi | 0.53 |
|  |  |  | Mohamed | Houseni | 0.22 |
|  |  |  | Zakaria | Mohamed | 0.40 |
|  |  |  | Tarik | Mohamed | 0.42 |
|  |  |  | Latifa | Mohamed Abdelgawad | 0.42 |
|  |  |  | Abde El-Galil | Amr | 0.43 |
|  | Algeria | 54 (0.6) | Ahmed | Bedir | 0.47 |
|  |  |  | Amira Yasmine | Benmelouka | 0.61 |
|  |  |  | Ammar | Elakhdar | 0.42 |
|  |  |  | Said | Grace | 0.37 |
|  |  |  | Yasmine | Hezema | 0.42 |
|  |  |  | Mohamed | Houta | 0.57 |
|  |  |  | Ahmed | Aioub | 0.43 |
|  |  |  | Mohamed | Lebda | 0.49 |
|  |  |  | Salim | Si-Mohamed | 0.61 |
|  |  |  | Mohamed | Balaha | 0.42 |
| Mexico |  |  |  |  |  |
|  | Spain | 4845 (82.6) | Roberto | Castro-Muñoz | 0.84 |
|  |  |  | Jorge | Corona-Castuera | 0.79 |
|  |  |  | José Eleazar | Aguilar-Toalá | 0.89 |
|  |  |  | José Luis | Maldonado-García | 0.94 |
|  |  |  | Juan | Monribot-Villanueva | 0.99 |
|  |  |  | Ivan | Ramos-Martínez | 0.80 |
|  |  |  | Juan Carlos | Rendón-Angeles | 0.99 |
|  |  |  | Rubén | Silva-Tinoco | 0.33 |
|  |  |  | José | Valero-Galván | 0.84 |
|  |  |  | Jorge | Velazquez-Roman | 0.74 |
|  | Portugal | 175 (3.0) | Juliana Santos | Batista-Oliveira | 0.79 |
|  |  |  | Patricia | Carmona-Levario | 0.42 |
|  |  |  | Luis | Fregoso-Aparicio | 0.37 |
|  |  |  | Hugo Virgilio | Perales-Vela | 0.52 |
|  |  |  | Hugo | Plascencia | 0.36 |
|  |  |  | Gisela | Rangel-Yescas | 0.39 |
|  |  |  | Carlos | Reséndiz-Aparicio | 0.43 |
|  |  |  | Hugo | Sobral | 0.89 |
|  |  |  | Dioselina Panamá | Tristán-Samaniego | 0.42 |
|  |  |  | Tania | López-Huante | 0.43 |
|  | Italy | 173 (3.0) | Martiniano | Bello | 0.62 |
|  |  |  | Giovanni | Carabali | 0.99 |
|  |  |  | Alberto | Cedro-Tanda | 0.42 |
|  |  |  | Elisa | Dorantes-Acosta | 0.42 |
|  |  |  | Gabriela | Fonseca-Camarillo | 0.52 |
|  |  |  | Marco Tulio | Angulo | 0.47 |
|  |  |  | Fabiola | Mendez-Arriaga | 0.42 |
|  |  |  | Marco Aurelio | Rendón-Medina | 0.43 |
|  |  |  | Adolfo | Andrade-Cetto | 0.66 |
|  |  |  | Andrea | Arango-Angarita | 0.43 |
|  | France | 109 (1.9) | Jade | Castellanos | 0.42 |
|  |  |  | Christian Haydeé | Flores-Balcázar | 0.53 |
|  |  |  | Yvonne | Flores-Medina | 0.40 |
|  |  |  | Jean Pierre | González-Gómez | 0.98 |
|  |  |  | Eric | Avila-Vales | 0.61 |
|  |  |  | Marie Catherine | Boll | 0.62 |
|  |  |  | Erick | Martínez-Herrera | 0.53 |
|  |  |  | Eric | Monterrubio-Flores | 0.77 |
|  |  |  | Berenice | Rivera-Paredez | 0.60 |
|  |  |  | Patricia | Tricorache | 0.55 |
|  | Ireland | 52 (0.9) | Patricia | Clark | 0.78 |
|  |  |  | Jimmy | Cojab | 0.42 |
|  |  |  | Blair | Darney | 0.69 |
|  |  |  | Norma | Flores-Holguín | 0.40 |
|  |  |  | Elizabeth | Lainez-Cerón | 0.58 |
|  |  |  | Erin | McKiernan | 0.92 |
|  |  |  | Dan | Morgenstern-Kaplan | 0.55 |
|  |  |  | Matthew | Reynolds | 0.84 |
|  |  |  | Teresa | Shamah-Levy | 0.72 |
|  |  |  | James | Thrasher | 0.53 |
| Pakistan |  |  |  |  |  |
|  | Pakistan | 6388 (93.8) | Ayaz | Ahmad | 0.93 |
|  |  |  | Muhammad | Irfan | 0.92 |
|  |  |  | Hafiz | Ahmed | 0.83 |
|  |  |  | Irfan | Ahmed | 0.86 |
|  |  |  | Rahat | Riaz | 0.92 |
|  |  |  | Aasma | Saeed | 0.89 |
|  |  |  | Dania Khalid | Saeed | 0.92 |
|  |  |  | Tania | Tamoor | 0.47 |
|  |  |  | Muhammad | Arshad | 0.94 |
|  |  |  | Aisha | Umar | 0.90 |
|  | Bangladesh | 110 (1.6) | Abdul | Hannan | 0.49 |
|  |  |  | Nazli | Hossain | 0.57 |
|  |  |  | Mohammad | Islam | 0.78 |
|  |  |  | Nayyer | Islam | 0.61 |
|  |  |  | Anmol | Mohan | 0.54 |
|  |  |  | Fazal | Rabbi | 0.33 |
|  |  |  | Saif Ur | Rahman | 0.45 |
|  |  |  | Nasir | Rahman | 0.45 |
|  |  |  | Siraj | Uddin | 0.62 |
|  |  |  | Noor | Ul Islam | 0.47 |
|  | India | 41 (0.6) | Kanti | Devi | 0.45 |
|  |  |  | Pankaj | Kumar | 0.93 |
|  |  |  | Ramesh | Kumar | 0.78 |
|  |  |  | Vinod | Kumar | 0.84 |
|  |  |  | Dileep | Kumar | 0.49 |
|  |  |  | Parma | Kumari | 0.46 |
|  |  |  | Om Prakash | Mehta | 0.58 |
|  |  |  | Vivaswan Dutt | Mishra | 0.90 |
|  |  |  | Jitander Kumar | Pabani | 0.65 |
|  |  |  | Rupesh | Raut | 0.78 |
|  | Malaysia | 33 (0.5) | Mohsina Noor | Ibrahim | 0.53 |
|  |  |  | Mohammad | Idris | 0.42 |
|  |  |  | Noor Ul | Ain | 0.56 |
|  |  |  | Noor Ul Huda | Maria | 0.55 |
|  |  |  | Rozina | Nuruddin | 0.42 |
|  |  |  | Adina | Rahim | 0.37 |
|  |  |  | Noor | Rahman | 0.52 |
|  |  |  | Faiqah | Ramzan | 0.60 |
|  |  |  | Nidaa | Harun | 0.59 |
|  |  |  | Shabina | Ariff | 0.44 |
|  | Egypt | 31 (0.5) | Ahmed | Donia | 0.76 |
|  |  |  | Ahmed Abdul | Habib | 0.54 |
|  |  |  | Hesham | Hamoda | 0.92 |
|  |  |  | Samina | Ismail | 0.36 |
|  |  |  | Osama | Mohiuddin | 0.63 |
|  |  |  | Rabab | Sakina | 0.42 |
|  |  |  | Maham | Salim | 0.48 |
|  |  |  | Azza | Warraitch | 0.42 |
|  |  |  | Lamia | Yusuf | 0.43 |
|  |  |  | Mona | Zulfikar | 0.43 |
| Indonesia |  |  |  |  |  |
|  | Indonesia | 2980 (77.9) | Michael | Dwinata | 0.53 |
|  |  |  | Adeodatus Yuda | Handaya | 0.83 |
|  |  |  | Agus Joko | Pitoyo | 0.99 |
|  |  |  | Arcellia Farosyah | Putri | 0.85 |
|  |  |  | Dwi Cahyani Ratna | Sari | 0.99 |
|  |  |  | Nydia Rena Benita | Sihombing | 0.87 |
|  |  |  | Sigit | Suharta | 0.94 |
|  |  |  | Tommy | Supit | 0.80 |
|  |  |  | Syahrida Dian | Ardhany | 0.90 |
|  |  |  | Daniel Joko | Wahyono | 0.85 |
|  | Malaysia | 178 (4.7) | Huda Shalahudin | Darusman | 0.74 |
|  |  |  | Khairuddin | Djawad | 0.42 |
|  |  |  | Achmad Kemal | Harzif | 0.42 |
|  |  |  | Rafli Zulfa | Kamil | 0.87 |
|  |  |  | Khairiah | Khairiah | 0.73 |
|  |  |  | Nur | Laili | 0.63 |
|  |  |  | Mohammad Zen | Rahfiludin | 0.59 |
|  |  |  | Hamzah | Shatri | 0.42 |
|  |  |  | Adrian Ujin | Yap | 0.75 |
|  |  |  | Muhammad Khifzhon | Azwar | 0.42 |
|  | Pakistan | 128 (3.3) | Mohammad | Iqbal | 0.90 |
|  |  |  | Muhammad | Kamil | 0.71 |
|  |  |  | Mohammad Rizki | Akbar | 0.62 |
|  |  |  | Muhammad | Aldika Akbar | 0.67 |
|  |  |  | Mudatsir | Mudatsir | 0.80 |
|  |  |  | Muhammad Mansyur | Romi | 0.54 |
|  |  |  | Safwan | Safwan | 0.48 |
|  |  |  | Nadira | Vadaq | 0.59 |
|  |  |  | Muhammad | Zulfajri | 0.73 |
|  |  |  | Yuriz | Bakhtiar | 0.42 |
|  | Italy | 52 (1.4) | Diana | Barsasella | 0.42 |
|  |  |  | Nico | Gamalliel | 0.42 |
|  |  |  | Adrianna | Bella | 0.67 |
|  |  |  | Armina | Fariani | 0.42 |
|  |  |  | Maria | Gayatri | 0.45 |
|  |  |  | Leonardus Yodi | Giovanni | 0.35 |
|  |  |  | Mara | Ipa | 0.43 |
|  |  |  | Gita Addelia | Nevara | 0.39 |
|  |  |  | Adriana | Palimbo | 0.43 |
|  |  |  | Gina | Saptiani | 0.43 |
|  | Ireland | 45 (1.2) | Dalla | Doohan | 0.84 |
|  |  |  | Jeannie | Flynn | 0.92 |
|  |  |  | Nyoman | Golden | 0.36 |
|  |  |  | Benjamin | Hegarty | 0.88 |
|  |  |  | Jeremias | Ivan | 0.42 |
|  |  |  | Nurdjannah Jane | Niode | 0.43 |
|  |  |  | Daniell Edward | Raharjo | 0.42 |
|  |  |  | Alan | Soffan | 0.59 |
|  |  |  | Kevin | Baird | 0.63 |
|  |  |  | Charles | Lisnahan | 0.58 |
| Nigeria |  |  |  |  |  |
|  | Nigeria | 2553 (75.8) | Oluwasomidoyin Olukemi | Bello | 0.97 |
|  |  |  | Oluwadamilola | Adejumo | 0.99 |
|  |  |  | Kikelomo Adebanke | Kolawole | 0.99 |
|  |  |  | Andrew | Olagunju | 0.49 |
|  |  |  | Adebunmi | Olarinoye | 0.99 |
|  |  |  | Abimbola | Oluyori | 0.99 |
|  |  |  | Sd | Osagie-Eweka | 0.60 |
|  |  |  | Ogadimma | Arisukwu | 0.99 |
|  |  |  | Osariyekemwen | Uyi | 0.99 |
|  |  |  | Ayansina | Ayanlade | 0.91 |
|  | Ghana | 139 (4.1) | Prosper Obed | Chukwuemeka | 0.42 |
|  |  |  | Richard | Aborisade | 0.54 |
|  |  |  | Solomon | E Owumi | 0.39 |
|  |  |  | Isaac | Adedara | 0.39 |
|  |  |  | Juliana | Afemikhe | 0.43 |
|  |  |  | Joseph | Agbaji | 0.42 |
|  |  |  | Ediomo-Ubong Ekpo | Nelson | 0.42 |
|  |  |  | Rose Okwunu | Abah | 0.59 |
|  |  |  | Larry | Awo | 0.42 |
|  |  |  | Mabel Kamweli | Aworh | 0.76 |
|  | Kenya | 107 (3.2) | Chris | Kwaja | 0.46 |
|  |  |  | Stanley | Njaka | 0.52 |
|  |  |  | Julius Beneoluchi | Odili | 0.47 |
|  |  |  | Kenneth | Omabe | 0.46 |
|  |  |  | Beryl | Ominde | 0.94 |
|  |  |  | Victor | Omoni | 0.48 |
|  |  |  | Richard | Onalo | 0.61 |
|  |  |  | Michael | Oraebosi | 0.42 |
|  |  |  | Gideon Adamu | Shallangwa | 0.43 |
|  |  |  | Stephen Chiahemba | Aondoakaa | 0.87 |
|  | Pakistan | 94 (2.8) | Umar Ali | Bukar | 0.92 |
|  |  |  | Muhammad Lawal | Abubakar | 0.60 |
|  |  |  | Dauda Usman | Maryam | 0.92 |
|  |  |  | Abdullahi | Muhammad | 0.84 |
|  |  |  | Naziru Bashir | Mukhtar | 0.85 |
|  |  |  | Sagir | Mustapha | 0.43 |
|  |  |  | Idris Zubairu | Sadiq | 0.34 |
|  |  |  | Muhammad | Saliu | 0.59 |
|  |  |  | Bilal | Sulaiman | 0.44 |
|  |  |  | Yahaya | Usman | 0.42 |
|  | Niger | 68 (2.0) | Mohammed Dantani | Adamu | 0.48 |
|  |  |  | Babatunde | Hamza | 0.68 |
|  |  |  | Wada | Ibrahim | 0.61 |
|  |  |  | Kasimu Ghandi | Ibrahim | 0.42 |
|  |  |  | Abubakar Alhaji | Liman | 0.42 |
|  |  |  | Adamu Haruna | Mamman | 0.62 |
|  |  |  | Alhassan | Alhassan | 0.42 |
|  |  |  | Isma'ila Arzika | Mungadi | 0.42 |
|  |  |  | Rabia Salihu | Sa'id | 0.43 |
|  |  |  | Abdoul-Madjidou | Yacoubou | 0.69 |
| Iraq |  |  |  |  |  |
|  | Iraq | 270 (26.8) | Abbas Hashim | Abdulsalam | 0.86 |
|  |  |  | Jamal | Hasoon | 0.21 |
|  |  |  | Karzan | Hawrami | 0.73 |
|  |  |  | Salma Abbas | Al-Hadad | 0.72 |
|  |  |  | Ali Abdul Hussein | Al-Janabi | 0.90 |
|  |  |  | Mohammed Hadi Ali | Al-Jumaili | 0.77 |
|  |  |  | Hayder | Al-Kuraishy | 0.74 |
|  |  |  | Aaiad | Al-Rikabi | 0.36 |
|  |  |  | Israa Hussein | Ali | 0.42 |
|  |  |  | Rawezh | Salih | 0.74 |
|  | Saudi Arabia | 215 (21.4) | Israa Ghazi | Abdulhadi | 0.39 |
|  |  |  | Rawof | Al Tuma | 0.33 |
|  |  |  | Zaidon | Al-Aqbi | 0.44 |
|  |  |  | Haitham | Al-Mubarak | 0.61 |
|  |  |  | Manwar | Al-Naqqash | 0.47 |
|  |  |  | Mohammed Ibrahim | Aladul | 0.79 |
|  |  |  | Ahmed | Alahmar | 0.67 |
|  |  |  | Mohammed | Abdalqadir | 0.46 |
|  |  |  | Ma'an | Nayif | 0.49 |
|  |  |  | Samih | Odhaib | 0.40 |
|  | Pakistan | 193 (19.2) | Ammar Fadil | Abid | 0.74 |
|  |  |  | Sallal | Abid | 0.61 |
|  |  |  | Ali Haif | Abbas | 0.42 |
|  |  |  | Qutuba | Karwi | 0.42 |
|  |  |  | Farhad Ahmed | Khudhur | 0.63 |
|  |  |  | Eham Amer | Ali | 0.43 |
|  |  |  | Basheer Husham | Ali | 0.42 |
|  |  |  | Azhar Kareem | Razzaq | 0.93 |
|  |  |  | Nahla Muhammad | Saeed | 0.74 |
|  |  |  | Bushra | Shlla | 0.42 |
|  | Egypt | 123 (12.2) | Ahmed | Abdulwahab | 0.73 |
|  |  |  | Thair Abdel Lateef | Hassan | 0.48 |
|  |  |  | Samer | Hoz | 0.42 |
|  |  |  | Darya | Abdulateef | 0.42 |
|  |  |  | Ahmed | Kaftan | 0.55 |
|  |  |  | Dalia Ahmed | Kalef | 0.74 |
|  |  |  | Raghad | Mohammed | 0.46 |
|  |  |  | Muthanna | Mohammed | 0.42 |
|  |  |  | Rana Fadhil | Mousa | 0.65 |
|  |  |  | Wael Waleed | Mustafa | 0.42 |
|  | Syria | 49 (4.9) | Sawsan Mohammed | Kareem | 0.35 |
|  |  |  | Mohammed Khalid | Al-Atrash | 0.53 |
|  |  |  | Baraa | Al-Rahawe | 0.48 |
|  |  |  | Suha Haithem | Mohammed | 0.29 |
|  |  |  | Karwan Ali | Omar | 0.42 |
|  |  |  | Rasha Khalil Abduljalil | Alsaad | 0.43 |
|  |  |  | Basil | Saleh | 0.42 |
|  |  |  | Ata Omer | Salih | 0.42 |
|  |  |  | Mohammed Ali | Selo | 0.66 |
|  |  |  | Jamal Kareem | Shakor | 0.43 |
| Ethiopia |  |  |  |  |  |
|  | Ethiopia | 3671 (91.1) | Mulat Gebeyehu | Baye | 0.99 |
|  |  |  | Molla Yigzaw | Birhanu | 0.99 |
|  |  |  | Gizachew Worku | Dagnew | 0.99 |
|  |  |  | Nigatu Regassa | Geda | 0.99 |
|  |  |  | Gebremedhin | Haile | 0.99 |
|  |  |  | Tamirat | Mamo | 0.95 |
|  |  |  | Ayenew | Mose | 0.86 |
|  |  |  | Weldegebrial | Aregawi | 0.97 |
|  |  |  | Gashaw Garedew | Woldeamanuel | 0.99 |
|  |  |  | Biniyam | Ayele | 0.95 |
|  | Egypt | 36 (0.9) | Hussen | Ebrahim | 0.69 |
|  |  |  | Hanan | Ahmed | 0.63 |
|  |  |  | Ismael | Ahmed | 0.42 |
|  |  |  | Abdulmuneim | Ahmed | 0.47 |
|  |  |  | Hanan | Ahmed | 0.63 |
|  |  |  | Hussen | Mohammed | 0.47 |
|  |  |  | Solomon Ahmed | Mohammed | 0.43 |
|  |  |  | Mohammed Aliy | Mohammed | 0.55 |
|  |  |  | Hussein | Mohammed | 0.53 |
|  |  |  | Mohammed Ahmed | Teka | 0.52 |
|  | Pakistan | 26 (0.7) | Muhammed | Haji | 0.60 |
|  |  |  | Muhammed | Hamid | 0.66 |
|  |  |  | Ammas Siraj | Mohammed | 0.73 |
|  |  |  | Annissa | Muhammed | 0.43 |
|  |  |  | Husniya Yasin | Amane | 0.43 |
|  |  |  | Riyaz Ahmad | Rather | 0.61 |
|  |  |  | Anwar | Shakir | 0.67 |
|  |  |  | Jafer | Siraj | 0.53 |
|  |  |  | Abrar | Sualeh | 0.42 |
|  |  |  | Menbeu | Sultan | 0.42 |
|  | Niger | 25 (0.6) | Fufa Dawo | Bari | 0.43 |
|  |  |  | Batala | Barsisa | 0.46 |
|  |  |  | Badhaasaa | Bayissa | 0.45 |
|  |  |  | Makka | Adam | 0.94 |
|  |  |  | Wario | Galma | 0.42 |
|  |  |  | Ali | Maalim Issack | 0.50 |
|  |  |  | Oumer | Ali | 0.22 |
|  |  |  | Haji Aman | Deybasso | 0.71 |
|  |  |  | Abdella | Aman | 0.58 |
|  |  |  | Sadikalmahdi | Abdella | 0.42 |
|  | Namibia | 18 (0.5) | Teshita Uke | Chikako | 0.42 |
|  |  |  | Tona Zema | Diddana | 0.42 |
|  |  |  | Seth | Inzaule | 0.29 |
|  |  |  | Ebisa Olika | Keyata | 0.42 |
|  |  |  | Debelo | Shikuro | 0.43 |
|  |  |  | Kegnie | Shitu | 0.44 |
|  |  |  | Shuma Gosha | Kanfe | 0.43 |
|  |  |  | Kaleegziabher | Lukas | 0.46 |
|  |  |  | Sewunet Sako | Shagaro | 0.42 |
|  |  |  | Tolassa | Ushula | 0.45 |
| Bangladesh |  |  |  |  |  |
|  | Bangladesh | 1955 (78.5) | Moin Uddin | Ahmed | 0.65 |
|  |  |  | Md Mahmudul Hasan | Khan | 0.98 |
|  |  |  | Sharful Islam | Khan | 0.94 |
|  |  |  | Ajoy | Kumer | 0.90 |
|  |  |  | Md Sultan | Mahmud | 0.96 |
|  |  |  | Md Sekendar | Ali | 0.74 |
|  |  |  | Mohammad Mojibur | Rahman | 0.94 |
|  |  |  | Iqbal | Sarker | 0.78 |
|  |  |  | Shaikh Jamal | Uddin | 0.59 |
|  |  |  | Sabina | Yeasmin | 0.84 |
|  | Pakistan | 307 (12.3) | Mirza | Hasanuzzaman | 0.39 |
|  |  |  | Syed A K Shifat | Ahmed | 0.83 |
|  |  |  | Hafsa Binte | Kibria | 0.53 |
|  |  |  | Zubair | Akhtar | 0.92 |
|  |  |  | Muhammed | Amanat | 0.79 |
|  |  |  | Wasim | Sabbir | 0.58 |
|  |  |  | Rahagir | Salekeen | 0.48 |
|  |  |  | Mohammad Sharif | Ullah | 0.69 |
|  |  |  | Habib | Ullah | 0.82 |
|  |  |  | Sabina | Yasmin | 0.42 |
|  | India | 64 (2.6) | Arun | Bose | 0.60 |
|  |  |  | Arka Jyoti | Chakraborty | 0.64 |
|  |  |  | Amit | Das | 0.42 |
|  |  |  | Goutam Kumar | Acherjya | 0.43 |
|  |  |  | Preeti | Jain | 0.74 |
|  |  |  | Satyajit | Kundu | 0.59 |
|  |  |  | Chandan | Sarkar | 0.48 |
|  |  |  | Haribondhu | Sarma | 0.55 |
|  |  |  | Sitesh | Bachar | 0.43 |
|  |  |  | Arpan | Baidya | 0.42 |
|  | Egypt | 22 (0.9) | Ahmed | Hossain | 0.43 |
|  |  |  | Mostafa | Kamal | 0.85 |
|  |  |  | Abdulla-Al | Kafy | 0.43 |
|  |  |  | Sabrina Samad | Shoily | 0.43 |
|  |  |  | Ibrahim | Khalil | 0.42 |
|  |  |  | Islam Md | Meftaul | 0.44 |
|  |  |  | Mohammed | Mohi-Ud-Din | 0.52 |
|  |  |  | Sayed Mohammad | Mohsin | 0.49 |
|  |  |  | Sara | Nowreen | 0.33 |
|  |  |  | Ahmed | Sharif | 0.65 |
|  | Saudi Arabia | 19 (0.8) | Faruq | Abdulla | 0.42 |
|  |  |  | Fahad | Jubayer | 0.74 |
|  |  |  | Faisal | Ahmmed | 0.42 |
|  |  |  | Hassan | Al-Emran | 0.42 |
|  |  |  | Mohammad | Al-Forkan | 0.45 |
|  |  |  | Marzia | Al-Hakeem | 0.45 |
|  |  |  | Murtuza | Al-Mueed | 0.43 |
|  |  |  | Mohammed | Nooruzzaman | 0.43 |
|  |  |  | Faisal | Omar | 0.75 |
|  |  |  | Omar | Sharif | 0.55 |
| Viet Nam |  |  |  |  |  |
|  | Viet Nam | 1842 (94.0) | Duy | Do | 0.98 |
|  |  |  | Trinh Thi My | Duyen | 0.99 |
|  |  |  | Thuan Duc | Lao | 0.99 |
|  |  |  | Nui Nguyen | Minh | 0.99 |
|  |  |  | Minh Tri | Ngo | 0.99 |
|  |  |  | Thi Kieu Loan | Nguyen | 0.99 |
|  |  |  | Thuan Thi | Nguyen | 0.99 |
|  |  |  | Bac | Nguyen Hoai | 0.99 |
|  |  |  | Van Han | Pham | 0.99 |
|  |  |  | Le Nguyen | Vu | 0.99 |
|  | Iran | 19 (1.0) | Mohammad | Ghalambaz | 0.77 |
|  |  |  | Naghmeh | Niknejad | 0.99 |
|  |  |  | Rasool | Pelalak | 0.61 |
|  |  |  | Behrooz | Keshtegar | 0.99 |
|  |  |  | Amirhosein | Mosavi | 0.99 |
|  |  |  | Mehrbakhsh | Nilashi | 0.82 |
|  |  |  | Yasser | Vasseghian | 0.51 |
|  |  |  | Meisam | Babanezhad | 0.95 |
|  |  |  | Meisam | Babanezhad | 0.95 |
|  |  |  | Meisam | Babanezhad | 0.95 |
|  | Ireland | 10 (0.5) | Joseph | Donovan | 0.92 |
|  |  |  | Susan | Dorman | 0.82 |
|  |  |  | Barnaby | Flower | 0.61 |
|  |  |  | Barnaby | Flower | 0.61 |
|  |  |  | Kimberly | Green | 0.50 |
|  |  |  | Kimberly Elizabeth | Green | 0.41 |
|  |  |  | Angela | McBride | 0.86 |
|  |  |  | Lisa | Redwood | 0.53 |
|  |  |  | David | Saakian | 0.29 |
|  |  |  | John | Wain | 0.74 |
|  | India | 7 (0.4) | Suraj Kumar | Bhagat | 0.74 |
|  |  |  | Raja | Das | 0.44 |
|  |  |  | Archana | Patel | 0.75 |
|  |  |  | Ponnurengam Malliappan | Sivakumar | 0.54 |
|  |  |  | Ponnurengam Malliappan | Sivakumar | 0.54 |
|  |  |  | Vinay Bharadwaj | Tatipamula | 0.74 |
|  |  |  | Vinay Bharadwaj | Tatipamula | 0.74 |
|  | Netherlands | 7 (0.4) | Cam | Van T Do | 0.38 |
|  |  |  | Thirumalaisamy | Velavan | 0.42 |
|  |  |  | Thirumalaisamy | Velavan | 0.42 |
|  |  |  | Thirumalaisamy | Velavan | 0.42 |
|  |  |  | Thirumalaisamy | Velavan | 0.42 |
|  |  |  | Thirumalaisamy | Velavan | 0.42 |
|  |  |  | Rogier | van Doorn | 0.92 |
| Tunisia |  |  |  |  |  |
|  | Tunisia | 1224 (75.0) | Haifa | Bichiou | 0.64 |
|  |  |  | Faiez | Boughanmi | 0.91 |
|  |  |  | Imene | Handous | 0.55 |
|  |  |  | Maha | Jamoussi | 0.62 |
|  |  |  | Emna | Jarrar | 0.82 |
|  |  |  | Najla | Jouini | 0.81 |
|  |  |  | Ichraf | Kraoua | 0.58 |
|  |  |  | Leila | Riahi | 0.71 |
|  |  |  | Emna Hidoussi | Sakly | 0.98 |
|  |  |  | Ghroubi | Sameh | 0.41 |
|  | Algeria | 149 (9.1) | Soulef | Bouaafia | 0.60 |
|  |  |  | Nadia | Bouattour | 0.42 |
|  |  |  | Farah | Chouchene | 0.42 |
|  |  |  | Nadia | Chérif | 0.37 |
|  |  |  | Ismail | Dergaa | 0.42 |
|  |  |  | Rahma | Guedri | 0.42 |
|  |  |  | Chahida | Harizi | 0.53 |
|  |  |  | Omar | Maamouri | 0.39 |
|  |  |  | Amira | Zaouaka | 0.42 |
|  |  |  | Nouha Ben | Abdeljelil | 0.43 |
|  | Morocco | 133 (8.2) | Amal | Boukteb | 0.43 |
|  |  |  | Khaoula | Chabbouh | 0.42 |
|  |  |  | Mehdi | Hasnaoui | 0.42 |
|  |  |  | Oumayma | Khayrallah | 0.52 |
|  |  |  | Sana | M'hir | 0.42 |
|  |  |  | Mouna | Ouaz | 0.42 |
|  |  |  | Nozha | Raguema | 0.45 |
|  |  |  | Mohamed Marouane | Saoudi | 0.43 |
|  |  |  | Mohamed | Tarroum | 0.42 |
|  |  |  | Khawla | Zinelabidine | 0.48 |
|  | Egypt | 37 (2.3) | Alaa | Bessadok | 0.42 |
|  |  |  | Sameh | Elkribi-Boukhris | 0.79 |
|  |  |  | Asma | Kassab | 0.59 |
|  |  |  | Sameh | Kaziz | 0.73 |
|  |  |  | Yosra | Mabrouk | 0.42 |
|  |  |  | Marwa | Mahmoudi | 0.37 |
|  |  |  | Asma | Saad | 0.72 |
|  |  |  | Mariem | Saada | 0.33 |
|  |  |  | Abdel Mlak | Said | 0.61 |
|  |  |  | Asma | Awadi | 0.42 |
|  | Niger | 14 (0.9) | Rahma | Goussi | 0.48 |
|  |  |  | Walid Sabri | Hamadou | 0.48 |
|  |  |  | Houda | Ilahi | 0.42 |
|  |  |  | Rekik | Issam | 0.31 |
|  |  |  | Bouhani | Malek | 0.39 |
|  |  |  | Amina | Amara | 0.42 |
|  |  |  | Salma | Mani | 0.28 |
|  |  |  | Msolli | Mohamed Amine | 0.42 |
|  |  |  | Idoudi | Safa | 0.48 |
|  |  |  | Abdoul Kader | Tapsoba | 0.42 |
| Kenya |  |  |  |  |  |
|  | Kenya | 665 (56.0) | Edwine | Barasa | 0.81 |
|  |  |  | Agnes | Langat | 0.66 |
|  |  |  | Loice | Mbogo | 0.73 |
|  |  |  | Dorah Kawira | Muthee | 0.99 |
|  |  |  | Sharon | Mwagira-Maina | 0.67 |
|  |  |  | Jackline | Oluoch-Aridi | 0.73 |
|  |  |  | Micah | Omare | 0.91 |
|  |  |  | Kenneth Otieno | Onditi | 0.99 |
|  |  |  | Prisca | Oria | 0.48 |
|  |  |  | Mercy | Wamalwa | 0.92 |
|  | Nigeria | 81 (6.8) | Jeremiah | Abolade | 0.46 |
|  |  |  | Adeyemi | Akinyemi | 0.97 |
|  |  |  | Oluwaseyi | Shorinola | 0.99 |
|  |  |  | Lukoye | Atwoli | 0.43 |
|  |  |  | Faith | Ebhodaghe | 0.44 |
|  |  |  | Adeniyi Francis | Fagbamigbe | 0.65 |
|  |  |  | Aderemi Timothy | Adeleye | 0.99 |
|  |  |  | Ifedayo | Adetifa | 0.95 |
|  |  |  | Bukola Babatunde | Adetokun | 0.99 |
|  |  |  | Frances | Adiukwu | 0.69 |
|  | Ireland | 57 (4.8) | Jason | Brotherton | 0.48 |
|  |  |  | Travis | Guy | 0.36 |
|  |  |  | Andrew | Karlyn | 0.40 |
|  |  |  | Megan | McHenry | 0.85 |
|  |  |  | Kara | Stuart | 0.53 |
|  |  |  | Beth | Tippett Barr | 0.78 |
|  |  |  | Harry | Wells | 0.49 |
|  |  |  | Kate | Wilson | 0.66 |
|  |  |  | Terrance | Baker | 0.65 |
|  |  |  | Brian | Bartilol | 0.51 |
|  | Congo | 43 (3.6) | Patrick | Bisimwa | 0.87 |
|  |  |  | Jacob | Iteba | 0.42 |
|  |  |  | Catherine | Kafu | 0.74 |
|  |  |  | Arthur Musakulu | Kemoli | 0.42 |
|  |  |  | Juvenal | Djangwani | 0.42 |
|  |  |  | Arnold | Lambisia | 0.42 |
|  |  |  | Adelaide | Lusambili | 0.42 |
|  |  |  | Raphael | Nyaruaba | 0.42 |
|  |  |  | Benjamin | Nyilitya | 0.42 |
|  |  |  | Felix | Bahati | 0.59 |
|  | Tanzania | 28 (2.4) | Kenneth | Juma | 0.43 |
|  |  |  | Pamela | Juma | 0.58 |
|  |  |  | Hassan | Kayondo | 0.42 |
|  |  |  | Beatrice | Madeghe | 0.42 |
|  |  |  | Felicita | Omari | 0.52 |
|  |  |  | Nyabinwa | Pascal | 0.51 |
|  |  |  | Sheila | Shaibu | 0.74 |
|  |  |  | Veronica | Shiroya | 0.46 |
|  |  |  | Marwa | Shumo | 0.42 |
|  |  |  | Tecla | Temu | 0.88 |
| Morocco |  |  |  |  |  |
|  | Morocco | 1091 (70.6) | Zineb | Ben Khadda | 0.39 |
|  |  |  | Kaoutar | Dib | 0.62 |
|  |  |  | Assiya | El Kettani | 0.67 |
|  |  |  | Mohamed | El Yaagoubi | 0.91 |
|  |  |  | Kaoutar | Elfazazi | 0.88 |
|  |  |  | Issam | Jandou | 0.45 |
|  |  |  | Rachida | Naciri | 0.74 |
|  |  |  | Mouna | Ouadghiri | 0.62 |
|  |  |  | Kerdoud | Ouassime | 0.42 |
|  |  |  | Khalid | Quertite | 0.44 |
|  | Algeria | 180 (11.7) | Zahira | Belattmania | 0.66 |
|  |  |  | Abdelhafid | Benksim | 0.42 |
|  |  |  | Yassamine | Bentata | 0.43 |
|  |  |  | Samia | Berrichi | 0.42 |
|  |  |  | Omar | Bouhnik | 0.54 |
|  |  |  | Oumaima | Boutoub | 0.37 |
|  |  |  | Mohamed | Bouzroud | 0.48 |
|  |  |  | Soufiane | Hamida | 0.43 |
|  |  |  | Ouassime | Kerdoud | 0.42 |
|  |  |  | Yasmine | Slimani | 0.42 |
|  | Niger | 49 (3.2) | Falmata Laouan | Brem | 0.94 |
|  |  |  | Oumniya | Abouhanine | 0.48 |
|  |  |  | Zakariya | Ghalmane | 0.50 |
|  |  |  | Ali | Ikrou | 0.52 |
|  |  |  | Salissou | Iro | 0.79 |
|  |  |  | Amina | Lissaneddine | 0.40 |
|  |  |  | Mouna | Ababou | 0.45 |
|  |  |  | Oussama | Anane | 0.34 |
|  |  |  | Zaina | Kadri | 0.42 |
|  |  |  | Najoua | Aballa | 0.36 |
|  | Tunisia | 47 (3.0) | Ichrak | Benamri | 0.47 |
|  |  |  | Abir | Chahouri | 0.48 |
|  |  |  | Sabrine | Derqaoui | 0.42 |
|  |  |  | Samir | Fakhour | 0.26 |
|  |  |  | Chaker | Kaoutar | 0.46 |
|  |  |  | Jawhar | Laamech | 0.42 |
|  |  |  | Moez | Amri | 0.92 |
|  |  |  | Anis | Sfendla | 0.43 |
|  |  |  | Samia | Arifi | 0.42 |
|  |  |  | Ahmed | Ballati | 0.42 |
|  | Egypt | 41 (2.7) | Matine | Abdelmalek | 0.48 |
|  |  |  | Hussam | Bouaamlat | 0.43 |
|  |  |  | Ahmed | Abed | 0.43 |
|  |  |  | El-Ouady | Fadwa | 0.44 |
|  |  |  | Ahmed | Ibrahimi | 0.43 |
|  |  |  | Mohamed | Labied | 0.41 |
|  |  |  | Gamal | Al Ati | 0.42 |
|  |  |  | Ahmed | Nafis | 0.53 |
|  |  |  | Hammam | Rasras | 0.43 |
|  |  |  | Mohamed | Zamd | 0.48 |
| Nepal |  |  |  |  |  |
|  | India | 476 (35.9) | Devi Prasad | Bhandari | 0.53 |
|  |  |  | Kiran | Acharya | 0.42 |
|  |  |  | Surakchhya | Gautam | 0.63 |
|  |  |  | Sumit | Agrawal | 0.90 |
|  |  |  | Mitesh | Karn | 0.48 |
|  |  |  | Vibha | Mahato | 0.42 |
|  |  |  | Narendra | Pandit | 0.56 |
|  |  |  | Gopal | Panta | 0.54 |
|  |  |  | Mangal | Rawal | 0.60 |
|  |  |  | Sudesh | Sharma | 0.69 |
|  | Nepal | 406 (30.6) | Rachana | Dhakal | 0.26 |
|  |  |  | Raju | Dhakal | 0.42 |
|  |  |  | Rajan | Ghimire | 0.61 |
|  |  |  | Sushil | Gyawali | 0.54 |
|  |  |  | Richa | Nepal | 0.47 |
|  |  |  | Prajwal | Paudel | 0.84 |
|  |  |  | Diptee | Poudel | 0.58 |
|  |  |  | Dipesh | Shakya | 0.60 |
|  |  |  | Deependra Kaji | Thapa | 0.43 |
|  |  |  | Subash | Thapa | 0.63 |
|  | Sri Lanka | 113 (8.5) | Buddha | Basnyat | 0.53 |
|  |  |  | Sunil | Basukala | 0.49 |
|  |  |  | Gehanath | Baral | 0.59 |
|  |  |  | Durga | Dhungana | 0.43 |
|  |  |  | Samikshya | Kandel | 0.42 |
|  |  |  | Suchana | Marahatta | 0.70 |
|  |  |  | Bandana | Shrestha | 0.34 |
|  |  |  | Kanchan | Thapa | 0.36 |
|  |  |  | Roshan | Timsina | 0.56 |
|  |  |  | Menuka | Aryal | 0.61 |
|  | Bangladesh | 82 (6.2) | Sapana | Bhujel | 0.42 |
|  |  |  | Sampanna | Chudal | 0.42 |
|  |  |  | Nashna | Maharjan | 0.37 |
|  |  |  | Anu | Maharjan | 0.32 |
|  |  |  | Sujen Man | Maharjan | 0.54 |
|  |  |  | Ashis | Pun | 0.58 |
|  |  |  | Komal Raj | Rijal | 0.51 |
|  |  |  | Sujit | Shah | 0.54 |
|  |  |  | Jyoti Tara Manandhar | Shrestha | 0.68 |
|  |  |  | Shyam Sharan | Shrestha | 0.42 |
|  | Indonesia | 55 (4.1) | Srijana | Adhikari | 0.27 |
|  |  |  | Anadi | Khatri | 0.42 |
|  |  |  | Buna | Bhandari | 0.44 |
|  |  |  | Hari Krishna | Laudari | 0.53 |
|  |  |  | Nurapati | Pantha | 0.78 |
|  |  |  | Malita | Amatya | 0.42 |
|  |  |  | Yuddha | Sapkota | 0.45 |
|  |  |  | Indra Prasad | Subedi | 0.67 |
|  |  |  | Prarthana | Subedi | 0.42 |
|  |  |  | Apurva Ratna | Tamrakar | 0.42 |
| Ghana |  |  |  |  |  |
|  | Ghana | 1036 (74.9) | Dorcas | Bredu | 0.94 |
|  |  |  | Emmanuel | Broni | 0.54 |
|  |  |  | Benedict | Calys-Tagoe | 0.76 |
|  |  |  | Cornelius | Dodoo | 0.84 |
|  |  |  | Moses | Aikins | 0.73 |
|  |  |  | Dickson Okoree | Mireku | 0.49 |
|  |  |  | Clement | Nyamekye | 0.60 |
|  |  |  | Daniel Atuah | Obeng | 0.99 |
|  |  |  | Reginald Adjetey | Annan | 0.99 |
|  |  |  | Ebenezer | Zaabaar | 0.84 |
|  | Ireland | 36 (2.6) | Robert Peter | Biney | 0.53 |
|  |  |  | Mochiah Moses | Brandford | 0.44 |
|  |  |  | Aaron | Christian | 0.49 |
|  |  |  | Benjamin Kingsley | Harley | 0.42 |
|  |  |  | Jerry Joe | Harrison | 0.89 |
|  |  |  | Frank | Hayford | 0.42 |
|  |  |  | Charles | Hayfron-Benjamin | 0.42 |
|  |  |  | William | Heve | 0.54 |
|  |  |  | Robert | Hinson | 0.52 |
|  |  |  | Rachel | Thompson | 0.58 |
|  | Kenya | 29 (2.1) | Mary Anti | Chama | 0.43 |
|  |  |  | John Kuumuori | Ganle | 0.67 |
|  |  |  | David | Adedia | 0.42 |
|  |  |  | Timothy Tienbia | Laari | 0.35 |
|  |  |  | Beatrice | Muriuki | 0.94 |
|  |  |  | Henry | Nagai | 0.38 |
|  |  |  | Charles | Noora | 0.55 |
|  |  |  | Charles Ochieng' | Olwal | 0.98 |
|  |  |  | Julius | Apidogo | 0.47 |
|  |  |  | Linus | Baatiema | 0.30 |
|  | Nigeria | 25 (1.8) | Akon | Ekpezu | 0.48 |
|  |  |  | Awolu | Adam | 0.64 |
|  |  |  | Alex Azuka | Ilechie | 0.74 |
|  |  |  | Bashiru Babatunde | Jimah | 0.96 |
|  |  |  | Sawyerr Louisa | Modupe | 0.57 |
|  |  |  | Emmanuella | Nzeribe | 0.80 |
|  |  |  | Michael | Olu-Taiwo | 0.69 |
|  |  |  | Oluwole | Owojuyigbe | 0.94 |
|  |  |  | Nkechi | Owoo | 0.76 |
|  |  |  | Oloche | Owoicho | 0.92 |
|  | Cameroon | 20 (1.5) | Ulric | Abonie | 0.42 |
|  |  |  | Alexandra Lindsey | Djomkam Zune | 0.96 |
|  |  |  | Julius | Dongdem | 0.66 |
|  |  |  | Valentina | Ngo-Bitoungui | 0.43 |
|  |  |  | Josiah Miner | Njem | 0.54 |
|  |  |  | Victor Fannam | Nunfam | 0.42 |
|  |  |  | Emmanuel | Kombiok | 0.47 |
|  |  |  | Omarine | Nlinwe | 0.35 |
|  |  |  | Waindim | Nyiambam | 0.72 |
|  |  |  | Benjamin | Thumamo Pokam | 0.81 |
| Philippines |  |  |  |  |  |
|  | Spain | 380 (34.1) | Angel | Bautista Vii | 0.83 |
|  |  |  | Maria Gay | Carrillo | 0.54 |
|  |  |  | Fides | Del Castillo | 0.96 |
|  |  |  | Mia Patrice | Dela Vega | 0.72 |
|  |  |  | Austin | Ferolino | 0.60 |
|  |  |  | Jose Antonio Ma | Garrido | 0.92 |
|  |  |  | Maria Tamara Isabelle | Manalo | 0.54 |
|  |  |  | Mar Aristeo | Poncio | 0.59 |
|  |  |  | Ma Anna Rita | Ramirez | 0.48 |
|  |  |  | Jessa | Ata | 0.43 |
|  | France | 140 (12.6) | Erika Marie | Bascos | 0.48 |
|  |  |  | Katherine Pia | Cabatbat | 0.43 |
|  |  |  | Trixia Anne | Co | 0.42 |
|  |  |  | Patricia Marie | Lusica | 0.65 |
|  |  |  | Joseph Christian | Obnial | 0.43 |
|  |  |  | Niñoval | Pacaol | 0.42 |
|  |  |  | Laurence Lloyd | Parial | 0.59 |
|  |  |  | Patricia | Simon | 0.31 |
|  |  |  | Isabelle Dominique | Tomacruz | 0.42 |
|  |  |  | Christine | de Guia | 0.67 |
|  | Ireland | 110 (9.9) | Dennice | Catambacan | 0.48 |
|  |  |  | Niamh | Corduff | 0.99 |
|  |  |  | John Mark | De Leon | 0.73 |
|  |  |  | Michelle Ann | Eala | 0.42 |
|  |  |  | Christopher | Lowbridge | 0.55 |
|  |  |  | Maryrose | Macaraan | 0.64 |
|  |  |  | Kim Gerald | Medallon | 0.53 |
|  |  |  | Ralph John Emerson | Molino | 0.43 |
|  |  |  | John Jamir Benzon | R Aruta | 0.42 |
|  |  |  | Michael | Tee | 0.48 |
|  | UK | 45 (4.0) | Jeff Clyde | Corpuz | 0.54 |
|  |  |  | Mark Jayson | Cortez | 0.46 |
|  |  |  | Rich Milton | Dulay | 0.62 |
|  |  |  | Christopher Ryan | Maboloc | 0.42 |
|  |  |  | Mark Donald | Reñosa | 0.46 |
|  |  |  | Sarah May | Sibug-Torres | 0.47 |
|  |  |  | Mark Louie | Ramos | 0.43 |
|  |  |  | Jed Martin | Tingson | 0.55 |
|  |  |  | Christian Wilson | Turalde | 0.47 |
|  |  |  | Grant | Singleton | 0.41 |
|  | Germany | 31 (2.8) | Jan Gresil | Kahambing | 0.36 |
|  |  |  | Scott | Meissner | 0.61 |
|  |  |  | Richultz | Montevirgen | 0.48 |
|  |  |  | Jennifer Ann | Wi | 0.27 |
|  |  |  | Michael | Bacus | 0.42 |
|  |  |  | Sandra Enn | Bahinting | 0.54 |
|  |  |  | Rich Ericson | King | 0.73 |
|  |  |  | Anton Heinrich | Rennesland | 0.95 |
|  |  |  | Julius | Tutor | 0.30 |
|  |  |  | Sandra Enn | Bahinting | 0.54 |
| Tanzania |  |  |  |  |  |
|  | Tanzania | 293 (43.5) | Jaffu | Chilongola | 0.69 |
|  |  |  | Ikunda | Dionis | 0.63 |
|  |  |  | Gasto | Frumence | 0.72 |
|  |  |  | Isaac | Lyatuu | 0.61 |
|  |  |  | Fatima | Mussa | 0.61 |
|  |  |  | Tumaini | Ndekero | 0.61 |
|  |  |  | Halfan | Ngowo | 0.92 |
|  |  |  | Festo | Shayo | 0.91 |
|  |  |  | Sophia Rogasian | Tairo | 0.42 |
|  |  |  | Mgeni | Tambwe | 0.78 |
|  | Kenya | 72 (10.7) | Doreen | Kamori | 0.65 |
|  |  |  | Offoro Neema | Kimambo | 0.34 |
|  |  |  | Geofrey | Makenga | 0.34 |
|  |  |  | Peter | Mangesho | 0.42 |
|  |  |  | Omary Mashiku | Minzi | 0.42 |
|  |  |  | Mucho | Mizinduko | 0.42 |
|  |  |  | Phares Gamba | Mujinja | 0.42 |
|  |  |  | Richard | Mwaiswelo | 0.42 |
|  |  |  | Nancy | Somi | 0.43 |
|  |  |  | Chacha Wambura | Werema | 0.93 |
|  | Congo | 41 (6.1) | Marceline | Finda | 0.42 |
|  |  |  | Esther | Kisanga | 0.42 |
|  |  |  | Chonge | Kitojo | 0.48 |
|  |  |  | Patrick | Mabula | 0.56 |
|  |  |  | Julie | Makani | 0.56 |
|  |  |  | Joseph | Massenga | 0.59 |
|  |  |  | Rachel | Masuke | 0.42 |
|  |  |  | Elias Vicent | Mayala | 0.43 |
|  |  |  | Innocent | Mboya | 0.42 |
|  |  |  | Joel Seme | Ambikile | 0.42 |
|  | Zimbabwe | 27 (4.0) | Joel | Changalucha | 0.42 |
|  |  |  | Peter | Chapa | 0.55 |
|  |  |  | Nyasiro | Gibore | 0.67 |
|  |  |  | Michael | Mahande | 0.42 |
|  |  |  | Clara | Chamba | 0.42 |
|  |  |  | Kenneth | Makata | 0.44 |
|  |  |  | Alphaxard | Manjurano | 0.32 |
|  |  |  | Victor Vicent | Matabura | 0.43 |
|  |  |  | Saul | Mpeshe | 0.42 |
|  |  |  | Brenda | Shewiyo | 0.35 |
|  | Pakistan | 25 (3.7) | Zulfiqarali | Abbas | 0.84 |
|  |  |  | Neelam | Ismail | 0.42 |
|  |  |  | Nadeem | Kassam | 0.42 |
|  |  |  | Sartaz | Begum | 0.60 |
|  |  |  | Rabia Abeid | Khaji | 0.65 |
|  |  |  | Farida | Ali | 0.48 |
|  |  |  | Rukhsar Shabir | Osman | 0.74 |
|  |  |  | Abid | Sadiq | 0.89 |
|  |  |  | Kauther | Musalam | 0.46 |
|  |  |  | Jamil | Suleiman | 0.48 |
| Cuba |  |  |  |  |  |
|  | Spain | 261 (85.6) | Guillermo Antonio | Barreto-Argilagos | 0.89 |
|  |  |  | Ibraín Enrique | Corrales-Reyes | 0.99 |
|  |  |  | Angel | Escobedo | 0.93 |
|  |  |  | Zoylen | Fernández-Fleites | 0.62 |
|  |  |  | Marité | García-Llano | 0.78 |
|  |  |  | Melaine | González-García | 0.48 |
|  |  |  | Mirelys Rodríguez | Alfaro | 0.95 |
|  |  |  | Yakdiel | Rodriguez-Gallo | 0.84 |
|  |  |  | Maribel | Vallespi | 0.90 |
|  |  |  | Dagoberto | Álvarez-Aldana | 0.48 |
|  | France | 9 (3.0) | Jomarien | García-Couce | 0.42 |
|  |  |  | René | Gato | 0.42 |
|  |  |  | Rady | Laborde | 0.48 |
|  |  |  | Rady | Laborde | 0.48 |
|  |  |  | Ivis | Moran-Bertot | 0.42 |
|  |  |  | Ivis | Moran-Bertot | 0.42 |
|  |  |  | Giselle | Pentón-Rol | 0.79 |
|  |  |  | Yamilé | Peña-Quián | 0.43 |
|  |  |  | Giselle | Autié-Castro | 0.57 |
|  | Portugal | 6 (2.0) | Lázaro Manuel | Filgueira | 0.38 |
|  |  |  | Magilé | Fonseca | 0.52 |
|  |  |  | Claudia | Miranda | 0.49 |
|  |  |  | Arlenis | Oliva | 0.47 |
|  |  |  | Luis Augusto | Piedra | 0.80 |
|  |  |  | Sandra | Rodríguez-Salgueiro | 0.42 |
|  | Italy | 4 (1.3) | Viviana Falcón | Cama | 0.42 |
|  |  |  | Roberto | Cañete | 0.39 |
|  |  |  | Liani | Coronado | 0.42 |
|  |  |  | Claudia | Morilla | 0.42 |
|  | Netherlands | 4 (1.3) | Roy | González-Alemán | 0.42 |
|  |  |  | Roy | González-Alemán | 0.42 |
|  |  |  | Gerry | Leisman | 0.42 |
|  |  |  | Gerry | Leisman | 0.42 |
